# Ovarian granulosa cell tumor characterization identifies FOXL2 as an immunotherapeutic target

**DOI:** 10.1172/jci.insight.136773

**Published:** 2020-08-20

**Authors:** Stefano Pierini, Janos L. Tanyi, Fiona Simpkins, Erin George, Mireia Uribe-Herranz, Ronny Drapkin, Robert Burger, Mark A. Morgan, Andrea Facciabene

**Affiliations:** 1 Department of Radiation Oncology and; 2 Ovarian Cancer Research Center, Perelman School of Medicine, University of Pennsylvania, Philadelphia, Pennsylvania, USA.

**Keywords:** Immunology, Oncology, Cancer immunotherapy

## Abstract

Granulosa cell tumors (GCT) are rare ovarian malignancies. Due to the lack of effective treatment in late relapse, there is a clear unmet need for novel therapies. Forkhead Box L2 (FOXL2) is a protein mainly expressed in granulosa cells (GC) and therefore is a rational therapeutic target. Since we identified tumor infiltrating lymphocytes (TILs) as the main immune population within GCT, TILs from 11 GCT patients were expanded, and their phenotypes were interrogated to determine that T cells acquired late antigen-experienced phenotypes and lower levels of PD1 expression. Importantly, TILs maintained their functionality after ex vivo expansion as they vigorously reacted against autologous tumors (100% of patients) and against FOXL2 peptides (57.1% of patients). To validate the relevance of FOXL2 as a target for immune therapy, we developed a plasmid DNA vaccine (FoxL2–tetanus toxin; FoxL2-TT) by fusing *Foxl2* cDNA with the immune-enhancing domain of TT. Mice immunization with FoxL2-TT controlled growth of FOXL2-expressing ovarian (BR5) and breast (4T1) cancers in a T cell–mediated manner. Combination of anti–PD-L1 with FoxL2-TT vaccination further reduced tumor progression and improved mouse survival without affecting the female reproductive system and pregnancy. Together, our results suggest that FOXL2 immune targeting can produce substantial long-term clinical benefits. Our study can serve as a foundation for trials testing immunotherapeutic approaches in patients with ovarian GCT.

## Abstract 2

FOXL2 may serve as a immunotherapeutic target for tumor infiltrating lymphocytes in ovarian granulosa cell tumors.

## Introduction

Granulosa cell tumors (GCT) of the ovary are rare tumors accounting for less than 5% of all ovarian malignancies. Due to the relatively high recurrence rate, 30% of women diagnosed with GCT will ultimately die 10–30 years after their initial diagnosis (1, 2). As GCT does not respond well to standard chemotherapy, novel therapeutic approaches are desperately needed.

Cancer immunotherapy aims to reprogram the patient’s immune system to fight its own cancer cells via recognition of tumor antigens. Immunotherapeutic agents can be divided into active and passive categories. Active immunotherapies, such as cancer vaccines, aim to instruct the immune system to recognize and attack tumor-associated antigens (TAAs) or tumor-specific antigens (TSAs) (3). Passive immunotherapies deal with adoptive transfer of ex vivo expanded cells (4) or exogenous administration of monoclonal antibodies. Adoptive T cell therapy (ACT) consists of isolating a cancer patient’s T cells, followed by selection (in case of tumor-infiltrating lymphocytes [TILs]) (5) or engineering (in case of CAR-T cells) (6), ex vivo expansion, and infusion back into the patient. ACT of TILs was first tested in melanoma patients and demonstrated impressive objective response rates of over 40% and a complete remission rate of up to 24% (7). Although cancer vaccines targeting nonself antigens (e.g., Cervarix, Gardasil9) have produced exciting results in the clinic (8), cancer vaccines encoding self TAA have shown poor efficacy, with only a small fraction of patients experiencing an objective clinical response (9, 10). Such limited efficacy is, in part, explained by the low immunogenicity of most TAAs, and accordingly, several strategies are being studied to boost immune responses. For example, in the case of plasmid-DNA cancer vaccines, in vivo electroporation increases DNA uptake, leading to enhanced antigen expression and concomitant increase in immune responses (11). Moreover, fusion of the antigen with the minimized domain of the C fragment of tetanus toxin (TT), has been used to elicit antigen-specific immune responses (12–14). Despite the limited efficacy of many clinical trials targeting self TAA, vaccines remain an attractive anticancer modality. They generally represent a specific, off-the-shelf intervention that is well tolerated and could lead to durable responses because of immunological memory.

Immunotherapy efficacy depends on T cell availability and TAAs they target. An ideal antigen for immunotherapy should be uniquely expressed by neoplastic cells, display robust antigenicity, and participate in key cellular functions to prevent the selection of malignant clones losing expression. Thus, the identification of potentially novel TAAs is critically important in exploring the potential of vaccines and ACT in cancer. Forkhead box protein L2 (FOXL2), a member of the forkhead-winged helix family, is a highly conserved transcription factor involved in virtually all stages of ovarian development and function (15–17). According to the Human Protein Atlas, FOXL2 protein is exclusively found in the ovary and the endometrium, while its RNA is also observed in endocrine tissues at a significantly lower level than female tissues (18). Indeed, substantial FOXL2 expression has been reported in the pituitary glands (19). Misregulation of FOXL2 expression and the presence of a highly recurrent somatic mutation C402G (C134W), identified in 90%–97% of GCT (1, 2), contributes to the transformation of normal granulosa cells to a malignant state (20). Interestingly, FOXL2 expression levels increase in GCT (20), as well as in some breast (21) and cervical cancers (22). High expression of FOXL2 correlates with worse overall survival in GCT (23), possibly due to its antiapoptotic role (24).

In this preclinical study, we characterized the immune landscape of GCT and identified T lymphocytes as the main immune population in the tumor microenvironment (TME). We successfully expanded TILs from 11 GCT patients and demonstrated that lymphocytes acquired late antigen-experienced memory phenotypes that express low PD1 levels. For those patients whose viable tumor cells were available, we tested their TIL reactivity and concluded that 9 of 9 patients had at least 1 TIL culture robustly reacting against autologous tumors. Seven patients were also tested for reactivity against FOXL2 peptides, and we demonstrated that 4 of them (57.1%) possessed FOXL2-specific TILs, suggesting that FOXL2 is an ideal target in GCT. With the goal of validating the immunogenicity and effectiveness of FOXL2-targeted response, we developed a plasmid-DNA vaccine encoding murine *Foxl2* that was able to reduce tumor progression in FOXL2-expressing ovarian and breast cancer models in a T cell–mediated manner. Combination of vaccination with anti–PD-L1 further suppressed tumor progression and improved mice survival without affecting female reproductive system and pregnancy.

## Results

### T lymphocytes is the main immune population within digested GCT.

**Figure 1:**
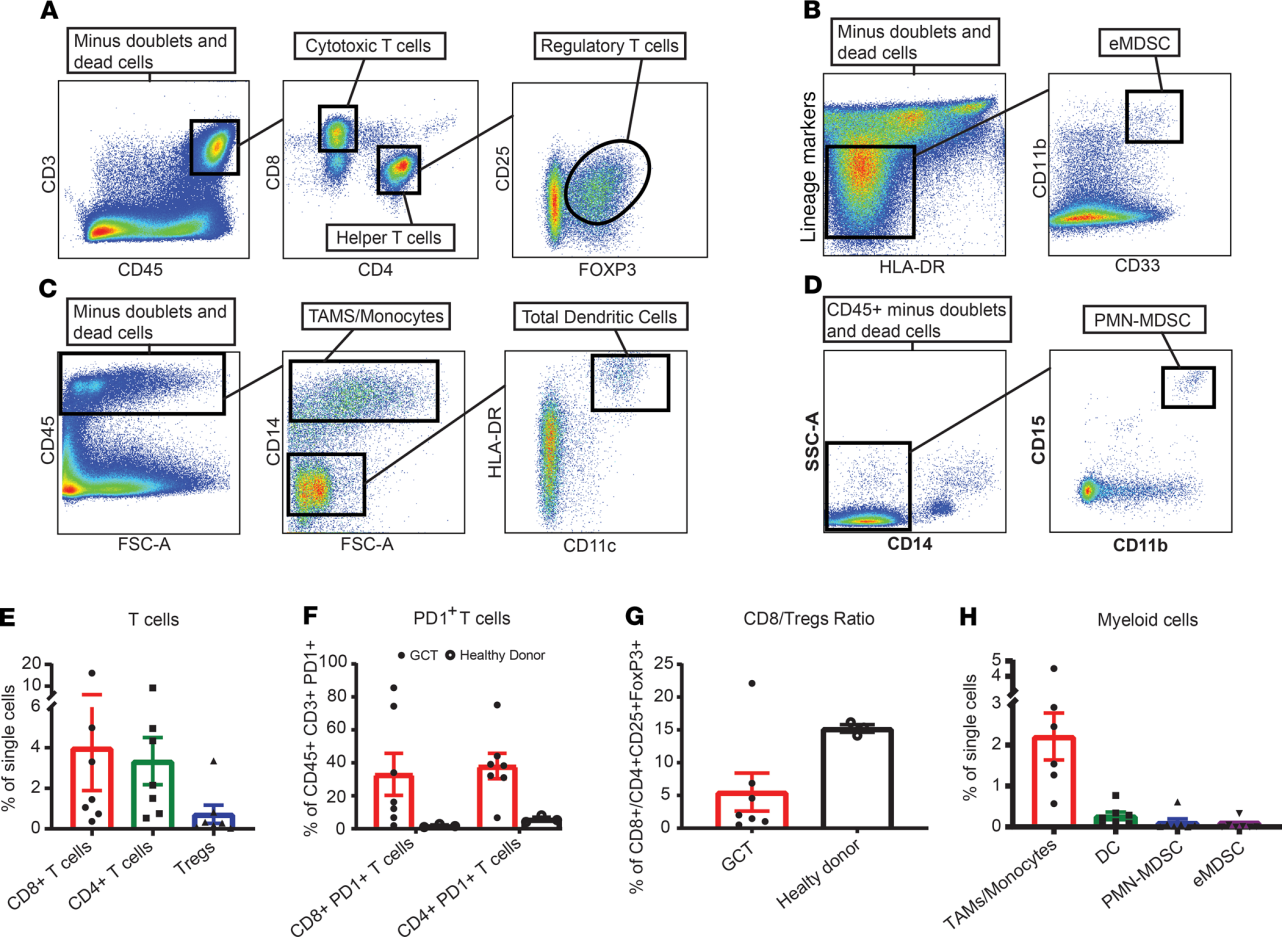
Lymphocytes make up the main immune population within digested GCT. Viable single tumor cell suspension and PBMCs from healthy donors were analyzed using polychromatic flow cytometry and progressive gating strategy. (**A**) Representative staining with CD3, CD4, CD8, CD25, CD45, and FOXP3 used to quantify helper (CD4^+^), cytotoxic (CD8^+^), and regulatory (Tregs) (CD4^+^CD25^+^FOXP3^+^) T cells in a GCT sample. (**B–D**) Representative staining with CD11b, HLA-DR, CD11c, Lineage, CD14, CD15, and CD33 used to identify the myeloid populations in a GCT sample. Tumor-associated macrophages (TAMs)/monocytes were separated from DC based on CD14 expression (**C**). Myeloid-derived suppressor cells (MDSC) were separated as eMDSC based on Lineage, HLA-DR, CD11b, and CD33 markers (**B**), whereas PMN-MDSC were characterized as CD15^+^CD14^–^CD11b^+^ (**D**). Proportions of tumor-infiltrating immune cells in GCT were quantified as percentage of total cell suspension. (**E**) Percentages of CD4^+^ T cells, CD8^+^ T cells, and Tregs compared with total tumor cell suspension. (**F**) Comparison of PD1-expressing T cells in the GCT vs. PBMCs. (**G**) CD8^+^ T cells/Tregs ratio in GCT vs. PBMCs. (**H**) Percentage of TAMs/monocytes, DC, PMN-MDSC, and eMDSC of total tumor cell suspension. Mean ± SEM is shown. Each dot represents a patient (*n* = 7) or healthy control PBMCs (*n* = 3).

The composition of tumor immune cell infiltration impacts the outcome of several human malignancies, as well as the response to anticancer therapies (25). In this study, we used multiparametric flow cytometry (Figure 1A) to quantify the number of helper (CD4^+^) and cytotoxic (CD8^+^) T cells as well as Tregs (CD4^+^CD25^+^FOXP3^+^) in GCT. We also develop a 9-color panel (Figure 1, B–D) to carefully characterize myeloid cells, such as tumor-associated macrophages (TAMs), DC, and myeloid-derived suppressor cells (MDSC). Peripheral blood mononuclear cells (PBMCs) from healthy donors were also included. Analyses of 7 GCT specimens showed that 4.0% of total tumor single cells suspensions were CD8^+^ T cells, 3.3% were CD4^+^ T cells and 0.72% were CD4^+^CD25^+^FOXP3^+^ Tregs (Figure 1E). Moreover, FACS staining indicated that both CD4^+^ and CD8^+^ T cells expressed increased levels of the activation marker PD1, which is suggestive of tumor-specific T cells (26, 27), compared with circulating T cells (CD8^+^PD1^+^ T cells; CD4^+^PD1^+^ T cells, *P* < 0.05) (Figure 1F). In ovarian cancer, it has been suggested that the effector/suppressor cell ratio may be a better indicator of outcome than individual T cell count (28). In ovarian GCT, we found a lower CD8^+^ T cells/Treg ratio than in healthy PBMCs (*P* = 0.067), likely contributing to an immunosuppressive tumor environment (Figure 1G). Our results also showed that TAMs/monocytes (CD45^+^CD14^+^) were the main myeloid population in GCT, accounting for 2.2% of total tumor single cell suspension (Figure 1H). DCs were separated from the TAMs/monocytes based on CD14, HLA-DR, and CD11c markers (29) (CD45^+^CD14^–^HLA-DR^+^CD11c^+^) and represented 0.27% of the total cell suspension. The MDSC populations (30) were marked as eMDSC (Lineage^–^CD11b^+^CD33^+^), amounting at 0.06%, and as PMN-MDSC (CD45^+^CD15^+^CD14^–^CD11b^+^), amounting at 0.11% of the total tumor cell suspension in GCT (Figure 1H). Using comparative real-time PCR, we observed a 16-fold increase of PD-L1 in flash-frozen GCT compared with PBMCs or with a non–GCT malignancy (renal cell carcinoma; RCC) (Supplemental Figure 3A; supplemental material available online with this article; https://doi.org/10.1172/jci.insight.136773DS1) (PBMCs vs. GCT, *P* = 0.05; non-GCT malignancy vs. GCT, not significant). In conclusion, our results show that GCT is significantly infiltrated by helper and cytotoxic lymphocytes, which are possibly tumor specific. However, the relatively high proportion of PD1^+^ T cells, CD8^+^ T cells/Treg ratio, and high TAMs/monocytes in the TME imply that GCT might establish immunosuppressive mechanisms to escape immune recognition.

### Memory phenotype TILs expressing a low level of PD1 compose the major subset after REP.

**Figure 2:**
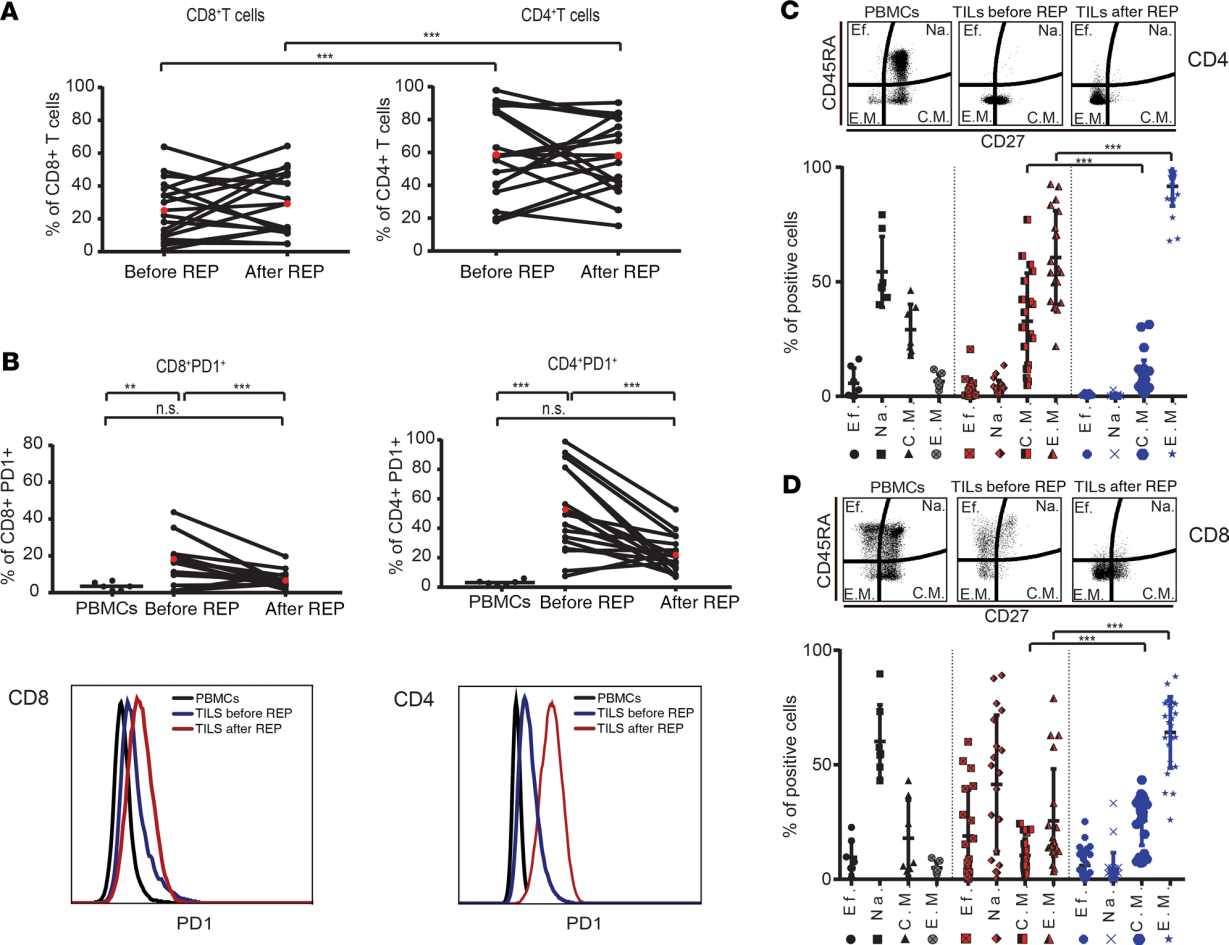
Memory phenotype TILs expressing low levels of PD1 is the major subset after rapid expansion protocol (REP). TILs were expanded from tumor fragments (1–2 mm^3^) of freshly resected GCTs and cultured in media containing IL-2. T cells before (pre-REP) and after REP (post-REP), as well as PBMCs from healthy donors, were stained, and percentage of T cell subtypes was estimated by flow cytometry. (**A**) Percentage of CD4^+^ TILs and CD8^+^ TILs before and after REP. Each bar represents TIL cultures from an independent tumor fragment. Red dots represent average; 10 patients were analyzed. (**B**) Percentages of PD1^+^CD8^+^ T cells and PD1^+^CD4^+^ T cells in pre- and post-REP (*n* = 10 patients) cultures, as well as in healthy PBMCs (*n* = 6 patients). (**C**) and (**D**) Percentage of T cells subtypes in pre- and post-REP (*n* = 10 patients) cultures and in healthy PBMCs (*n* = 6). Each point represents an independent TIL fragment (or culture). Mean ± SEM is shown. Tukey’s multiple comparison tests were performed. *P < 0.05, **P < 0.01, ***P < 0.001.

Most immunotherapies aim to boost the presence of tumor-reactive T cells within the solid tumor; therefore, we studied the feasibility of expanding T cells within GCT. Freshly resected tumors (*n* = 11) were minced into small fragments (~1–2 mm^3^) and plated as 1 fragment per well in media containing IL-2 (31). After approximately 3 weeks, wells with about 1 × 10^6^ cells were further expanded for 2 additional weeks using rapid expansion protocol (REP), allowing quick expansion of TILs to ~1 × 10^8^ cells (32) (post-REP TILs). All 11 GCT samples were successfully expanded, analyzed, and cryopreserved. FACS phenotype demonstrated that virtually all pre- and post-REP TILs were CD3^+^ (data not shown). The proportion of CD4^+^ T cells was significantly higher than CD8^+^ T cells in both pre-REP (*P* < 0.0001) and post-REP TIL samples (*P* = 0.0007) (Figure 2A). Furthermore, both CD4^+^ and CD8^+^ T cells had significantly higher levels of PD1 before REP than those of PBMCs (CD4^+^ T cell, *P* < 0.0001; CD8^+^ T cell, *P* = 0.0023), though levels dropped significantly after REP to became statistically indistinguishable (CD4, *P* = 0.15; CD8, *P* = 0.72) from PBMCs (Figure 2B) (PD1^+^CD4^+^ T cell pre-REP vs. post-REP, *P* = 0.0002; PD1^+^CD8^+^ T cell pre-REP vs. post-REP, *P* = 0.0007).

It has been demonstrated that memory phenotype TILs, specifically the central memory (T_CM_) subset, is required for effective ACT therapy (33). Surface expression of CD27 and CD45RA markers can be used to differentiate T cells into 4 subtypes: effector (T_E_, CD27^−^CD45RA^+^), naive (T_N_, CD27^+^CD45RA^+^), T_CM_ (CD27^+^CD45RA^−^), and effector memory (T_EM_, CD27^−^CD45RA^−^) (34). Similar to what has been reported (34), healthy PBMCs — used as control population — showed a typical distribution composed largely of T_N_, followed by T_CM_, T_E_, and hardly any T_EM_ (Figure 2, C and D). In GCTs, the CD4^+^ T cells in pre-REP TILs were predominantly memory T cells (32.8% T_CM_ and 60.6% T_EM_), which transitioned after-REP to become almost entirely T_EM_ (91.7%) (*P* < 0.0001) (Figure 2C). The CD8^+^ T cells in pre-REP TILs, however, showed a more heterogeneous phenotype (18.9% T_E_, 41.5% T_N_, 10.4% T_CM_, and 25.5% T_EM_) (Figure 2D). Similar to the CD4^+^ subset, the CD8^+^ T cells transitioned to a memory phenotype (25.7% T_CM_ and 64.1% T_EM_) during REP, but interestingly, both the T_CM_ and T_EM_ significantly increased after REP compared with pre-REP (CD8^+^ T_CM_, *P* < 0.0001; CD8^+^ T_EM_, *P* < 0.0001) (Figure 2D).

**Figure 3:**
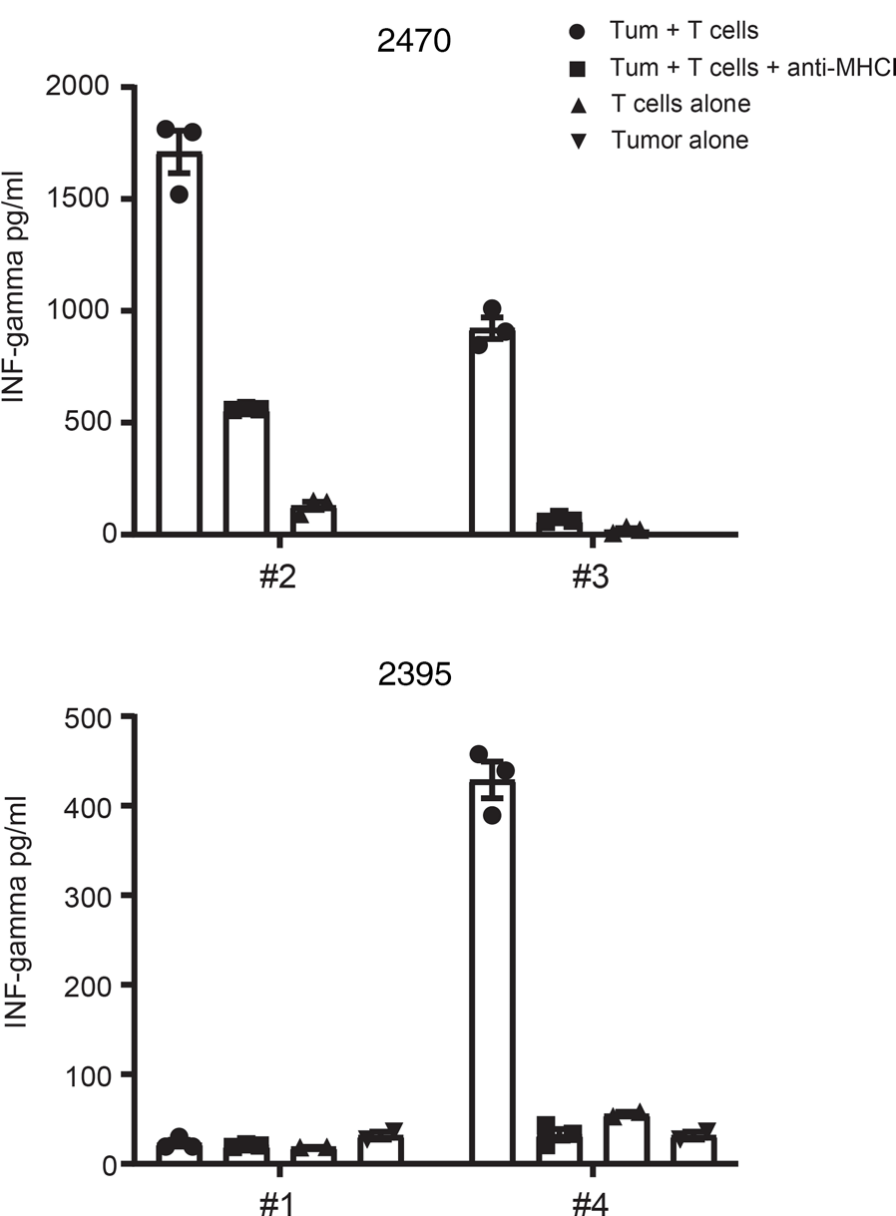
Expanded TILs recognize autologous tumors. A total of 3 × 10^5^ to 5 × 10^5^ of IL-2–rested TILs (post-REP) from individual cultures was coculture overnight with viable autologous tumor cells (CD45-depleted) at 1:1 ratio. Where class I blocking experiments were performed, anti–HLA-ABC was also added to tumor cells. T cell activation was assessed by measuring secreted IFN-γ in the supernatant using ELISA. The bar graphs illustrate the IFN-γ values from 2 representative patients showing 3 responding TILs fragments (2470 #2 and #3, and 2395 #4) and 1 unresponding fragment (2395 #1). Mean ± SD is shown.

Selection of TILs’ cultures for ACT requires screening of tumor-reactive T cells. To assess TIL reactivity, IL-2–rested TILs (post-REP) were cocultured overnight with autologous tumor cells, and ELISA was used to determine IFN-γ secretion, a marker of lymphocyte activation. Out of the 11 patients, only 9 had viable tumor cells to test T cell reactivity. Remarkably, all 9 patients tested had at least 1 TIL culture showing increased IFN-γ production after exposure to autologous tumor cells (Figure 3 and Supplemental Figure 1A). IFN-γ production was usually decreased with the addition of anti–MHC class I (anti-MHCI) blocking antibody, indicating that tumor recognition by TILs was MHCI mediated. For some samples, pre-REP TILs were also screened for tumor reactivity. Data from patient 2493 show that, after REP, IFN-γ production significantly increased compared with the pre-REP TILs, possibly due to a higher CD8^+^/CD4^+^ T cell ratio observed after REP in this sample (Supplemental Figure 1B). Ability to kill target cells was also tested using TILs and primary GCT cells both derived from patient 2522, the only patient whose tumor cells stably grew in vitro (Supplemental Figure 2).

Altogether, these results indicate that TILs from GCT can easily be expanded to large numbers, making them suitable for ACT approaches. After REP, the CD4^+^ TILs become entirely T_EM_, while the CD8^+^ counterpart maintain a pronounced T_CM_ phenotype, which is important for an effective ACT. Moreover, TILs decrease PD1 expression during REP and vigorously respond to autologous tumors, possibly demonstrating reversion from immune dysfunction to a more effective status.

### Expanded TILs recognize epitopes of FOXL2 protein.

**Figure 4:**
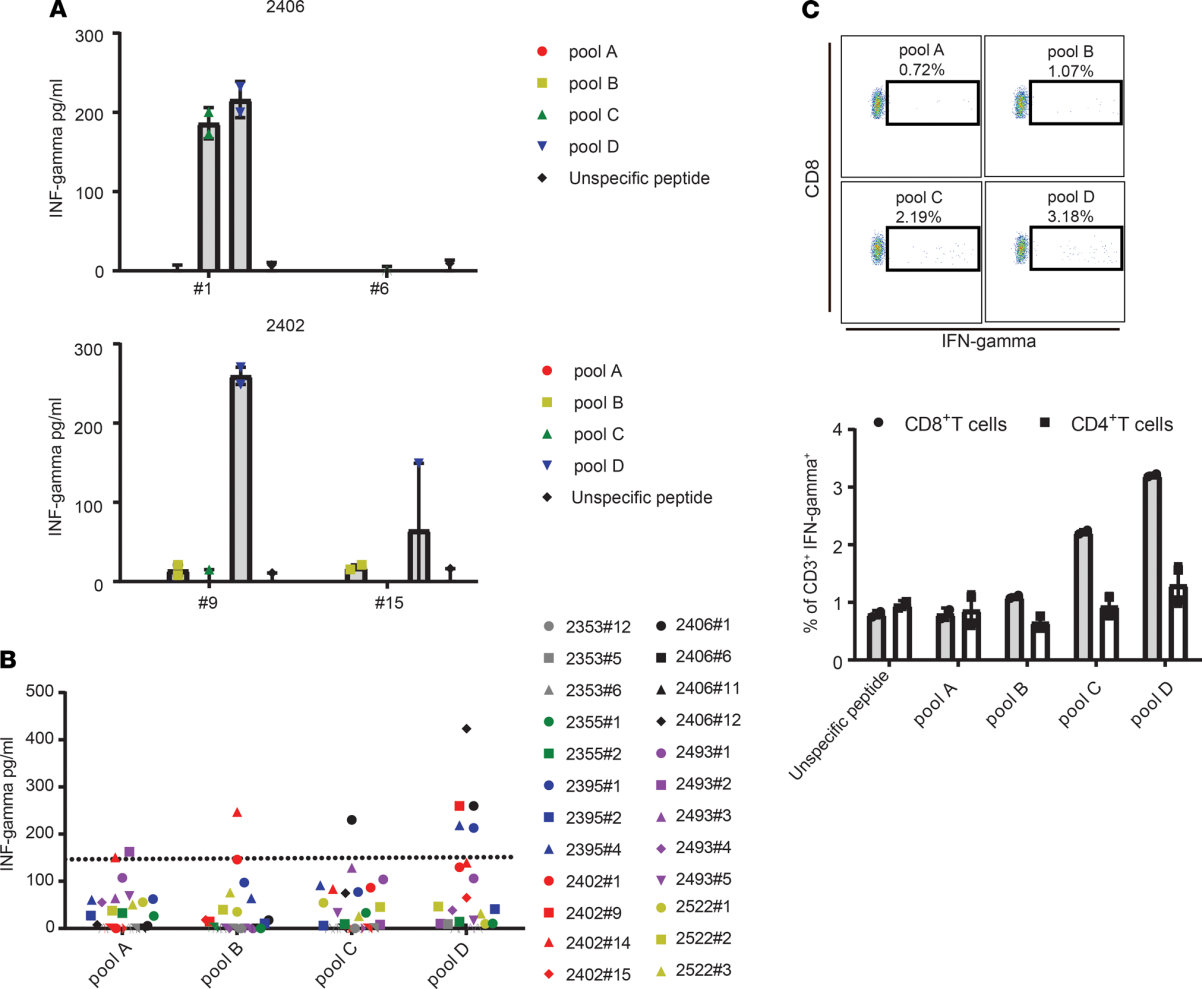
Expanded TILs recognize the GCT marker FOXL2. The human FOXL2 peptides library (91 peptides) was divided in 4 pools and used to assess TIL reactivity to FOXL2. A total of 1 × 10^5^ of IL-2 rested TILs (post-REP) from individual culture was cocultured overnight with autologous PBMCs pulsed with FOXL2 peptides at a 1:1 ratio. T cell activation was assessed using IFN-γ ELISA (**A** and **B**) and IFN-γ intracellular staining (ICS) (**C**). (**A**) Bar chart shows IFN-γ values from 2 representative patients with 2 responding fragments (2406 #1 and 2409 #9) and 2 unresponding fragments (2406 #6 and 2402 #15). (**B**) Chart shows the IFN-γ values divided by pool (*n* = 7 patients). A 150 pg/mL threshold was set to distinguish positive from negative values. (**C**) Representative FACS plots show IFN-γ ICS of specimen 2406 #1. Bar graph summarizes the ICS values from specimen 2406 #1. Mean ± SD is shown.

Since endogenous FOXL2 is upregulated in GCTs (Supplemental Figure 3B) and 90%–97% of GCTs contain a somatic mutation (FOXL2^C134W^) that can lead to the generation of a neoantigen, we investigated whether patients spontaneously possess reactive TILs against FOXL2. To test this hypothesis, we developed a FOXL2 peptide library encompassing the entire human FOXL2^C134W^ protein, including the mutation C134W, divided in 4 pools: pool A (sequences spanning aa 1–103), pool B (aa 93–195), pool C (aa 185–287), and pool D (aa 277–376). To this end, we measured IFN-γ production by ELISA and intracellular staining (ICS) after overnight coculture of IL-2–rested TILs with autologous PBMCs pulsed with FOXL2 peptides pools. As revealed in Figure 4, A and B, 7 patients were tested — those with available PBMCs — and 4 of them (57.1%) possessed at least 1 TIL fragment specific to FOXL2 pools. Moreover, IFN-γ ICS performed on sample 2406 #1 showed CD8^+^ T cells reacting against pool C and pool D (Figure 4C), validating the ELISA. Figure 4B summarizes the normalized values of TIL reactivity sorted by peptide pools. Because most patients recognized pool D, pool D was deemed a hot-spot region likely to contain immunodominant epitopes. Interestingly, limited reactivity was observed against pool B, which contained peptides covering the C134W mutation. Stimulation of PBMCs from healthy donors with the FOXL2 peptide library revealed a lack of T cell activation (data not shown), suggesting that, in physiological conditions, FOXL2 protein does not induce spontaneous adaptive immune response. Altogether, the results suggest that T cell–infiltrating GCTs are reactive against autologous tumors and that part of such reactivity is targeted toward FOXL2 epitopes.

### DNA immunization with FoxL2-TT breaks immune tolerance to FOXL2 in 3 different strains of mice.

**Figure 5:**
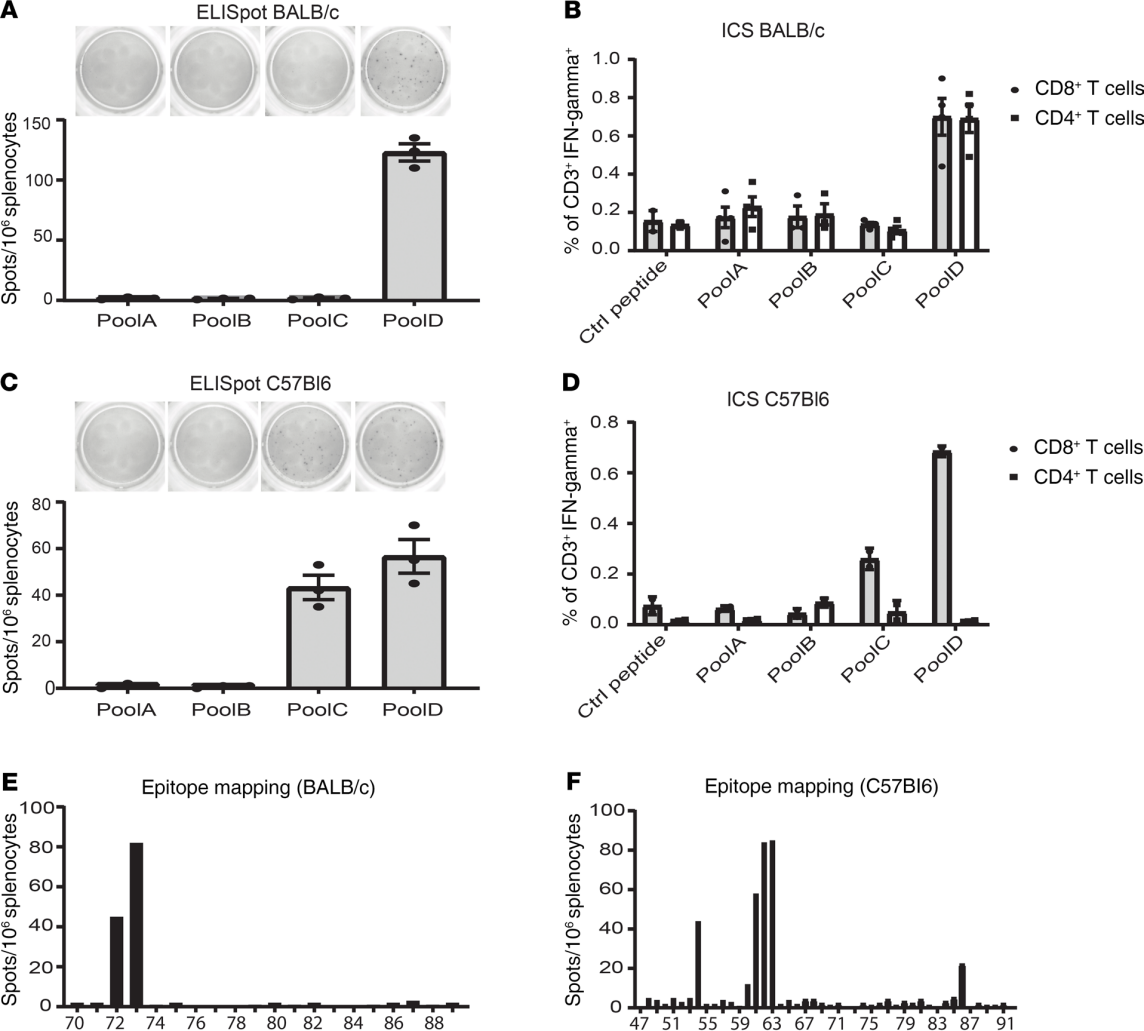
Vaccination with FoxL2-TT breaks the tolerance to FOXL2 protein. A total of 1 × 10^6^ splenocytes from FoxL2-TT vaccinated BALB/c or C57BL/6 mice was stimulated overnight with the mouse FOXL2 library or individual peptide and tested by ELISpot and ICS. Bar graphs illustrating number of IFN-γ spots (**A**, **C**, **E**, and **F**) show that BALB/c reacted against pool D (**A**) and against peptide 72 and 73 (**E**), whereas C57BL/6 reacted against both pool C and D (**C**) and against peptide 54, 61, 62, 63, and 86 (**F**). (**B** and **D**) Percentages of IFN-γ-secreting T cells show that both CD4^+^ and CD8^+^ T cells in BALB/c reacted upon overnight stimulation with pool D (**B**). Only CD8^+^ T cells reacted against pool C and pool D in C57BL/6 (**D**). Each data point represents a mouse. Mean ± SD is shown. Data are from 1 of 3 independent experiments.

To study the in vivo effectiveness of FOXL2 targeting, we developed a plasmid-DNA vaccine encoding murine FOXL2. Plasmid vaccines are closed circular DNA expression vectors designed to deliver antigens encoded under a strong promoter. Our DNA vaccine was generated using a pVAX plasmid vector that encoded the codon-optimized mouse *Foxl2*^C389G^ cDNA, which harbored the C130W mutation (corresponding to C134W in human) and was fused in frame with the first domain of the C fragment of the *TT* sequence (TT 865–1120), used as an immune enhancer (11, 14, 35) (Supplemental Figure 4A, left panel). Fusion of FOXL2 and TT was confirmed by Western blot (WB) (Supplemental Figure 4B). The resulting construct (FoxL2-TT) was injected 3 times at weekly intervals in healthy C57BL/6, BALB/c, and Tg (HLA-A2.1) mice. Priming/boost injections were followed by in vivo electroporation. One week after the last immunization, splenocytes harvested from vaccinated mice were stimulated with the mouse FOXL2 peptide library, consisting of pools A (aa 1–103), B (aa 93–195), C (aa 185–286), and D (aa 276–375). IFN-γ ELISpot indicated that the majority of T cell epitopes in BALB/c were contained within pool D (Figure 5A), whereas spleen-derived T cells from vaccinated C57BL/6 and Tg (HLA-A2.1) mice exhibited reactivity against both pools C and D (Figure 5C and Supplemental Figure 5A). IFN-γ ICS confirmed the ELISpot results and further showed that, in BALB/c, both CD4^+^ and CD8^+^ T cells reacted against pool D (Figure 5B), whereas CD8^+^ but not CD4^+^ T cells reacted against pools C and D in C57BL/6 (Figure 5D). FoxL2-TT immunization did not induce any FOXL2^C130W^-specific T cell response, as highlighted by the lack of reactivity against pool B, which contained the peptides harboring the C130W mutation. By individually using each single peptide from pools C and D, we found multiple immunodominant reactive peptides including #54 (FOXL2_213–227_); #61, #62, and #63 (FOXL2_241–255_, FOXL2_245–259_, and FOXL2_249–263_, respectively); and #86 (FOXL2_341–355_) in C57BL/6 mice, as well as peptide #72 (FOXL2_285–299_) and #73 (FOXL2_289-303_) in BALB/c mice, as demonstrated by ELISpot (Figure 5, E and F). These data were further validated by a MHCI binding prediction software (Supplemental Table 1) indicating ASYGPYSRV (FOXL2_249–257_), contained within peptides #62 and #63, as the best H-2Kb–restricted binder in C57BL/6. Finally, IFN-γ ICS showed that both CD4^+^ T cells and CD8^+^ T cells were activated by peptide #72 and #73, suggesting their binding to MHCI and MHCII in BALB/c (Supplemental Figure 5B). Together, these data confirm the immunogenicity of FOXL2 and indicate that it is possible to break the immune tolerance to FOXL2 in different animal strains and elicit T cells response.

### FoxL2-TT immunization suppresses tumor growth in FOXL2-expressing ovarian and breast cancer models.

**Figure 6:**
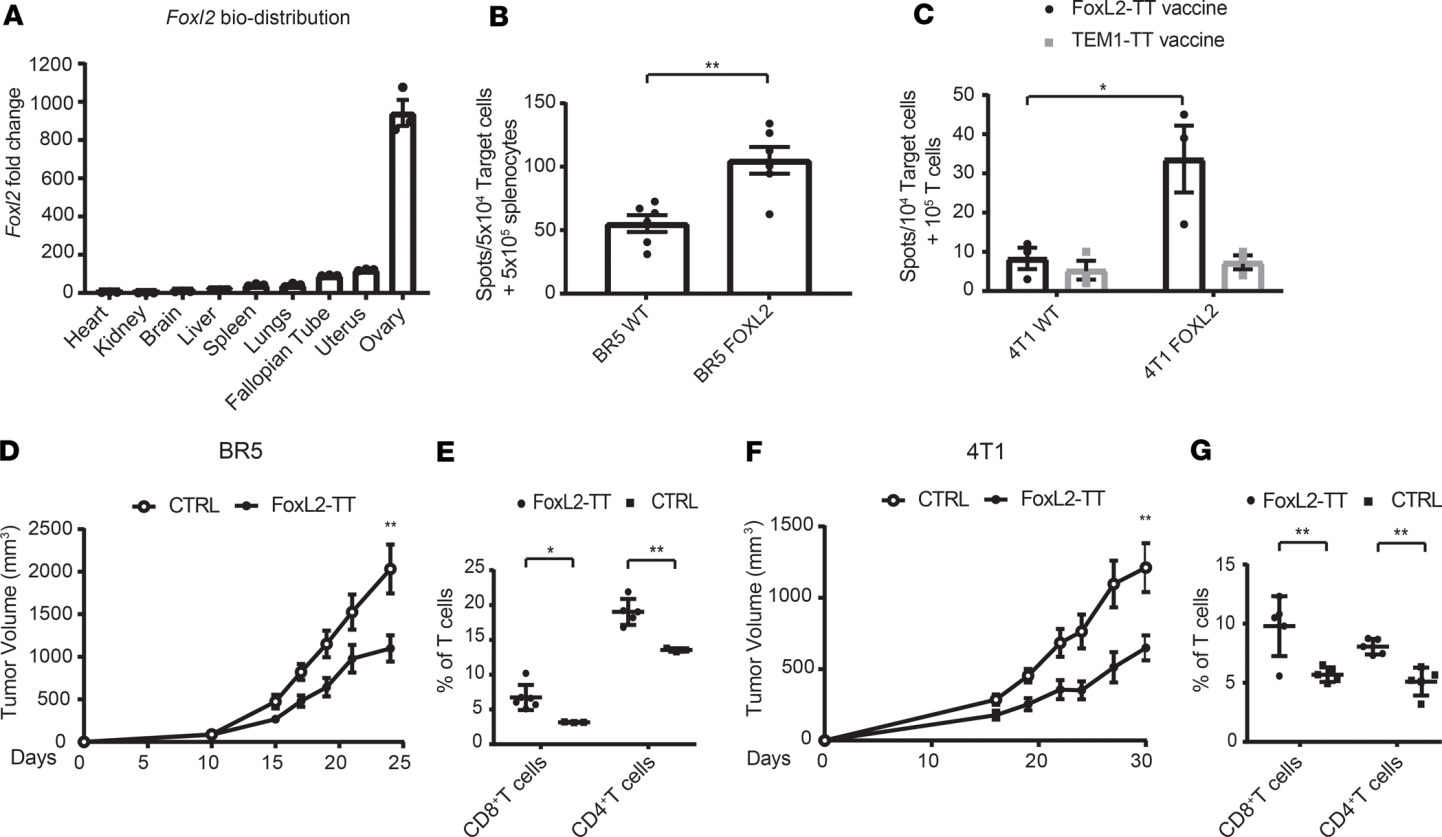
Vaccination with FoxL2-TT reduced tumor progression. (**A**) Healthy organs and tissues were collected from 3 mice, pooled together. RNA was extracted using Trizol, and *foxl2* fold expression was calculated by real-time PCR. Mean ± SEM is shown. (**B** and **C**) T cells from FVB and BALB/c vaccinated mice were cocultured with BR5-FOXL2 and BR5 WT (**B**) and with IFN-γ–pretreated 4T1-FOXL2 and 4T1 WT (**C**). (**D** and **F**) FVB and BALB/c were injected s.c. with 1 × 10^6^ BR5-FOXL2 (**D**) and 2.5 × 10^5^ 4T1-FOXL2 (**F**) and, 3 days later, injected 3 times with FoxL2-TT or empty pVAX (CTRL) vaccines followed by electroporation. Data are shown as mean ± SEM (*n* = 8–10 mice per group) from 1 of 3 experiments. Two-way ANOVA analyses were performed for tumor growth experiments. Percentage of tumor-infiltrating CD8^+^ and CD4^+^ T cells were calculated by flow cytometry at day 25. (**E** and **G**) Each dot represents a mouse, *n* = 6–3 mice per group (**E**) and *n* = 5 mice per group (**G**). Mean ± SEM is shown. Two-tailed *t* test analyses were performed. *P < 0.05, **P < 0.01.

Using Real-time PCR, we first measured the endogenous *Foxl2* expression in different mouse organs and found *Foxl2* mostly expressed in the ovary (Figure 6A), paralleling human data (Human Protein Atlas; ref. 18). Due to the lack of any suitable GCT model (36) to test in vivo vaccine’s efficacy, we attempted to identify FOXL2 expression in several tumor cell lines. In line with a FOXL2 expression pattern in human female tissues (18, 21), we found a very limited level of *Foxl2* cDNA in 2 ovarian cancer cell lines (BR5 and ID8) and 1 breast cancer cell line (4T1) (Supplemental Figure 6). The lack of *Foxl2* expression prompted us to overexpress mutated *Foxl2*^C389G^ in BR5 and 4T1 cancer cell lines using a pCMV6-A-PURO vector (Supplemental Figure 4A, right panel). Significant expression of FOXL2 was confirmed by WB and FACS (Supplemental Figure 4B and Supplemental Figure 4, C and D, left panels), and no difference in both in vitro (not shown) and in vivo tumor progression between the WT and the FOXL2-expressing cell lines was observed (Supplemental Figure 4, C and D, right panels). We then tested the specificity of vaccine-primed T cells to recognize FOXL2-expressing cells. To this end, we vaccinated FVB mice with FoxL2-TT, magnetically isolated T cells from splenocytes and exposed BR5-FOXL2 and BR5 WT to the T cells. IFN-γ ELISpot assay revealed that overexpression of FOXL2 in BR5 significantly increased T cell activation (*P* = 0.002) compared with BR5 WT (Figure 6B). Similarly, BALB/c mice were vaccinated with FoxL2-TT and with a control vector encoding an irrelevant antigen fused with TT (TEM1-TT plasmid-DNA vaccine; ref. 11). 4T1-FOXL2 and 4T1 WT cells were then exposed to T cells isolated from vaccinated mice. Results from Figure 6C show that T cells from FoxL2-TT–vaccinated mice, but not from TEM1-TT–vaccinated mice, recognized the 4T1-FOXL2 cells (*P* = 0.047) but not 4T1 WT parental cells.

To assess the in vivo antitumor effects of FOXL2 immunization, tumor cells were inoculated s.c., and 3–5 days later, mice were given 3 weekly vaccine injections followed by in vivo electroporation. Therapeutic FoxL2-TT vaccination suppressed tumor progression compared with control vector (4T1, *P* = 0.0062; BR5, *P* = 0.0028) in BR5-FOXL2 and 4T1-FOXL2 tumor–bearing mice (Figure 6, D and F) and improved mouse survival (BR5, *P* < 0.02; 4T1, *P* < 0.04) (Supplemental Figure 7). In line with in vitro results, vaccination of mice bearing BR5 WT failed to significantly slow down tumor progression (Supplemental Figure 8, A and B), indicating an antigen-specific effect of the vaccine. Analysis of the TME by flow cytometry revealed heavy infiltration of CD8^+^ and CD4^+^ T cells after vaccination in BR5-FOXL2 (CD8^+^, *P* < 0.02; CD4^+^, *P* < 0.003) (Figure 6E) and 4T1-FOXL2 (CD8^+^, *P* < 0.008; CD4^+^, *P* < 0.002) tumors (Figure 6G) versus control constructs. In the 4T1-FOXL2 model, we also studied the phenotypes (Supplemental Figure 9, A and B) of both bulk and FOXL2-reactive T cells, and we found that T_N_ concentrate in the periphery (spleen and lymph node [LN]) whereas T_EM_ accumulate within the tumor, mirroring our human T cell data. In contrast, FOXL2-reactive T cells were typically effector memory.

To determine if FoxL2-TT immune response was T cell mediated and to assess whether adoptive transfer of FOXL2-specific T cells would be efficacious in controlling growth of establish tumors, we performed ACT of T cells from immunized mice to recipient tumor–bearing mice. Transfer of CD3^+^ and CD8^+^ T cells was able to significantly suppress the progression of tumors expressing FOXL2, compared with control CD3^+^ T cells (4T1, CD3^+^ *P* < 0.001, CD8^+^ *P* = 0.012; BR5, CD3^+^ *P* < 0.02, CD8^+^ *P* = 0.05). Moreover, transfer of CD4^+^ T cells inhibited tumor progression in BR5-FOXL2, but not in 4T1-FOXL2, compared with control CD3^+^ T cells (4T1, nonsignificant; BR5 CD4^+^, *P* < 0.03) (Supplemental Figure 10, A and B).

Cumulatively, these results demonstrate the therapeutic impact of FOXL2-specific T cells in 2 tumor models expressing FOXL2. Moreover, ACT experiments also demonstrate that transfer of FOXL2-restricted T cells controls tumor progression, indicating a T cell–mediated effect of the vaccine.

### Combination of FoxL2-TT DNA immunization and anti–PD-L1 further suppresses tumor progression.

**Figure 7:**
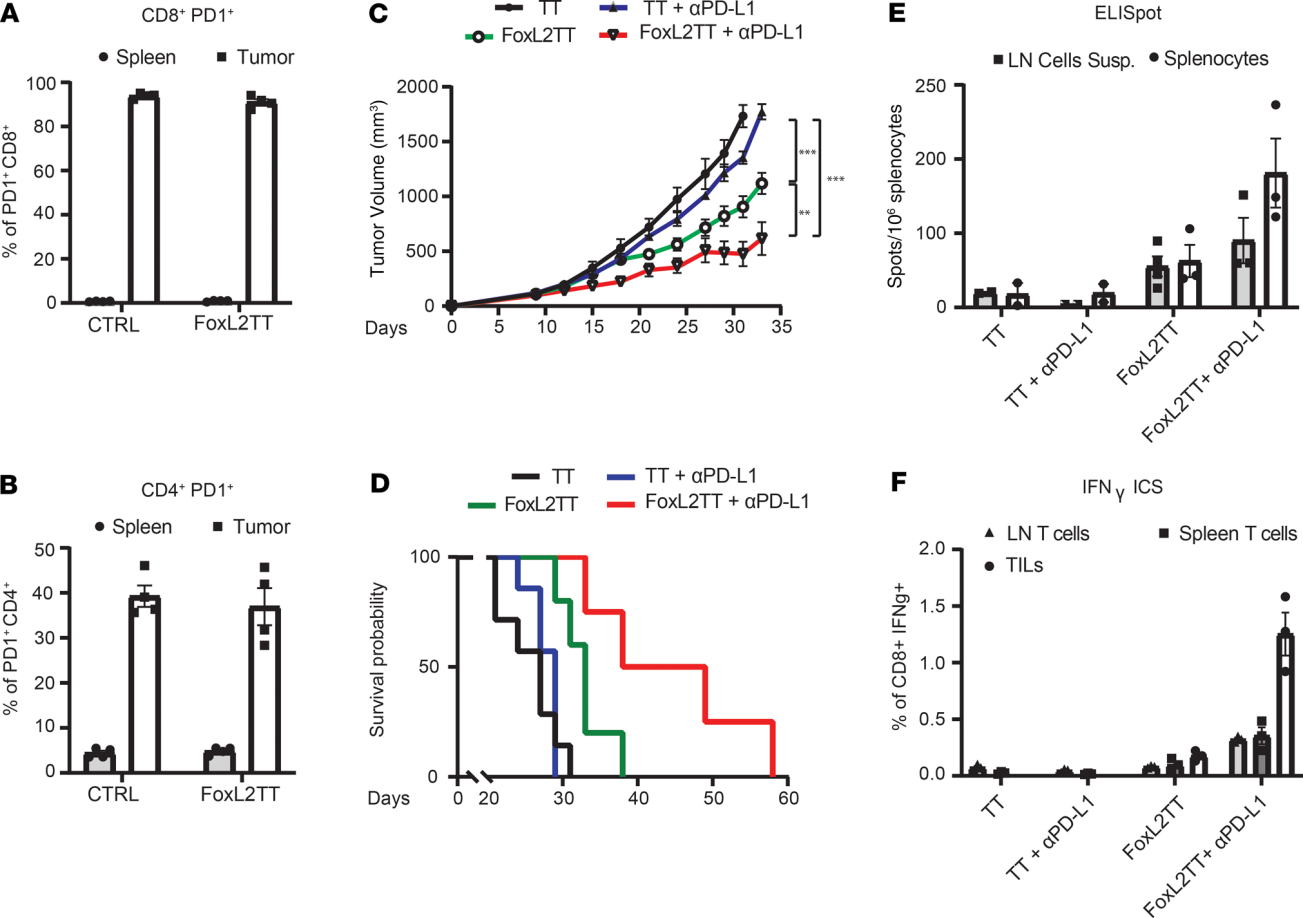
Combination of FoxL2-TT and anti–PD-L1 antibody further reduces tumor growth and improves mice survival. (**A** and **B**) T cells from spleen and tumor suspension were stained to assess PD1 expression level. Each data point represents 1 mouse. Mean ± SEM is shown. (**C** and **D**) BALB/c were injected s.c. with 2.5 × 10^5^ 4T1-FOXL2 and, 5 days later, injected 3 times with FoxL2-TT or TT vaccines followed by electroporation. Data are shown as mean ± SEM (*n* = 6–8 mice per group). Two-way ANOVA analyses were performed for tumor growth experiments (**C**). For the Kaplan-Meier analysis (**D**), cutoff values for tumor volume of 1000 mm^3^ for 4T1 were set to assess mice expiration. Long-rank test was performed to estimate statistical significance. (**E** and **F**) A total of 1 × 10^6^ cells (spleen, LN, and tumor suspension) from vaccinated BALB/c mice were stimulated overnight with BALB/c-reactive peptide #73 and tested by ELISpot (**E**) and ICS (**F**). Bar graphs illustrating number of IFN-γ spots (**E**) and percentage of IFN-γ–secreting cells (**F**). Each data point represents 1 mouse. Mean ± SEM is shown.

Although immunization with Foxl2-TT plasmid-DNA significantly reduced tumor progression, we hypothesized that the vaccine’s efficacy could be further improved via combination therapy. Since increased PD1 expression was observed both in TIL of human GCT (Figure 2B) and on mouse intratumor CD4^+^ and CD8^+^ T cells (Figure 7, A and B), we combined PD1/PD-L1 inhibition with FoxL2-TT vaccination in the 4T1-FOXL2 model. To this end, we followed the same immunization protocol described above, but this time, an anti–PD-L1 was administered for 4 times, every 3 days, starting at day 12. Mice receiving the combination of vaccine and anti–PD-L1 suppressed tumor progression (Figure 7C) (FoxL2-TT plus anti–PD-L1 vs. FoxL2-TT, *P* = 0.0042) and improved mice survival compared with vaccination (*P* < 0.007) or anti–PD-L1 (*P* < 0.001) monotherapies (Figure 7D). Combination therapy also increased FOXL2-restricted immune response in the spleen and LN (Figure 7E) and improved anti-FOXL2 T cell infiltration in the tumor (Figure 7F). In conclusion, adding anti–PD-L1 to FoxL2-TT vaccination significantly improved the antitumor effect and mice survival compared with monotherapy.

### FoxL2-TT DNA vaccination does not affect mouse reproductive system and pregnancy.

**Figure 8:**
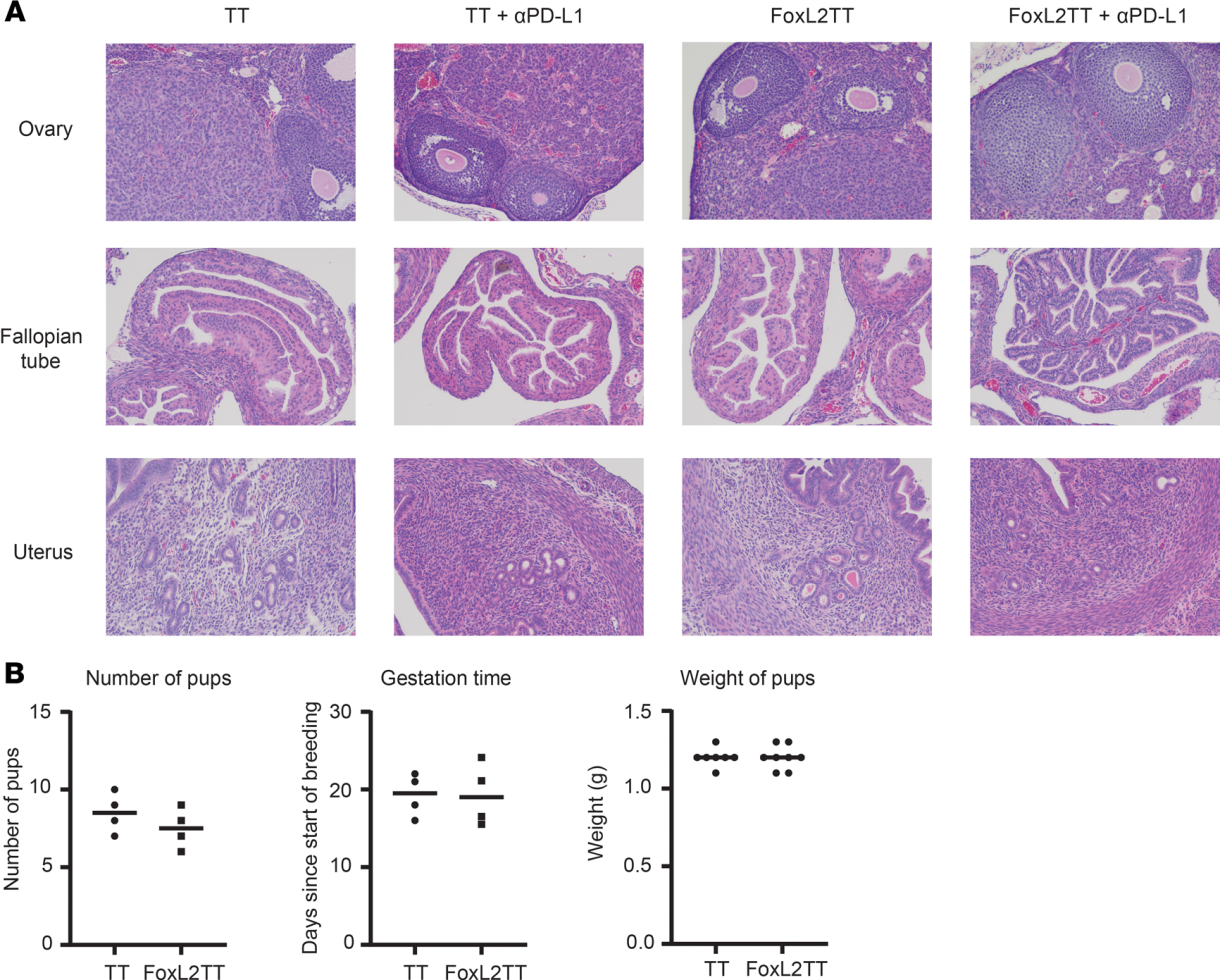
FoxL2-TT vaccination doesn’t affect pregnancy and reproduction. (**A**) BALB/c mice were tumor challenged with 4T1-FOXL2 and then vaccinated 3 times. One week after the last vaccination, the ovary, fallopian tube, and uterus were exported for H&E staining. Magnification, ×4. (**B**) Healthy BALB/c female mice were vaccinated 3 times and, 1 week after the last vaccination, allowed to mate with healthy males. Time to gestation, number of pups, and weight of pups at birth were followed over 4 cycles of pregnancy. Data are from 1 representative pregnancy. Each data point represents 1 mouse.

As FOXL2 expression is confined in the reproductive organs and inflammation influences pregnancy (37), we investigate whether FoxL2-TT immunization would affect female gestation. Female mice were immunized with FoxL2-TT or TT vaccine with or without anti–PD-L1. To assess potential inflammation and toxicity due to T cell activation, we performed H&E staining in the ovary, fallopian tubes, and uterus and found that vaccination does not induce inflammatory infiltrates, indicating that FOXL2-specific immune response is not directed to normal tissues (Figure 8A).

Embryo implantation and placentation require a careful immunological balance. Intrauterine inflammation (IUI) is strongly associated with preterm birth, with clinical evidence of inflammation in up to 40% of preterm births (37). Furthermore, exposure to IUI during prenatal development is a known risk factor for adverse neurodevelopmental outcomes in offspring (38). Therefore, to test the possible impact of FoxL2-TT immunization on pregnancy, female mice were immunized with FoxL2-TT or TT vaccine, allowed to bred with healthy males, and monitored during 4 cycles of pregnancy. No effects on time to gestation , total litter size, and pup weight at birth compared with control immunization were observed (Figure 8B) (11). Collectively, FoxL2-TT vaccination appears to have no untoward effects on the mouse reproductive system.

## Discussion

Because the immune system plays a critical role in tumor growth in virtually all solid tumors, exploring the immune landscape of GCT can guide development of novel immunotherapeutic approaches where effective treatment is lacking. The TME is composed of multiple immune cell types known to hamper lymphocyte-mediated attack, including Tregs, TAMs, and MDSCs. Several studies demonstrated that accumulation of Tregs and, in particular, a lower CD8^+^ T cell/Treg ratio within the tumor, correlates with poor clinical outcome in many cancers (39). In our GCT cohort, TILs were the most abundant immune cell type in the TME. In line with other malignancies (26), PD1 expression was consistently increased on TILs compared with T cells within PBMCs. The CD8^+^ T cell/Treg ratio in the tumor decreased compared with healthy PBMCs, suggesting that Tregs participate to the immunosuppressive environment of GCT. MDSCs are a heterogeneous cell population characterized by the ability to suppress T cells and NK cell function (40), as well as the ability to limit DC cross-presentation (41). Two studies in RCC (42) and colorectal cancer (43) found intratumor MDSCs to amount at about 5% and 2.99% of total tumor cell suspension, respectively. In our cohort of patients, the percentage of MDSCs was inferior to what was reported in RCC and colorectal cancer, underling the great disparity in the distribution and phenotypes of MDSCs in human cancers (29). TAMs represent diverse and heterogeneous populations of cells characterized by considerable plasticity. According to FACS analysis, they were the most prominent myeloid population in GCT. Finally, DC are the most powerful antigen-presenting cells and are critical in immune response initiation and development (44). It has been demonstrated that, although antigen presentation by DCs in the TME can be profoundly impaired (45), infiltration of tumors by DCs has often been linked to favorable prognosis in ovarian cancer (46) and other malignancies (47). DCs can be distinguished from TAMs based on low CD14 expression levels (29). In conclusion, our study shows that GCT is significantly infiltrated with multiple immune cell populations, likely affecting GCT responsiveness to therapies and patient outcomes.

ACT has shown remarkable efficacy, including overall response rates of 40% in metastatic melanoma, 90% in acute lymphoblastic leukemia, and 40% in chronic lymphoblastic leukemia (48). Because GCTs are heavily infiltrated by T cells, we investigated the feasibility of expanding TILs from freshly resected ovarian GCT. ACT efficacy depends upon both quantity and quality of the expanded lymphocytes. For instance, Radvanyi et al. demonstrated that the number of CD8^+^ T cells within the infused TILs are of critical importance in mediating tumor regression and increasing patient survival (49). However, the ratio between CD4: and CD8 in expanded TILs depends on the type of tumor as well as the culturing methods and can result in a heterogeneous ratio within the same study. For instance, while Dudley et al. (50) reported that most melanoma TILs cultures were predominantly CD8^+^, others showed no consistent frequency of CD4 and CD8 among the expanded cultures (51). In our study, expanded TILs were mostly CD4^+^ T cells, averaging to 58%, which is similar to pancreatic cancer according to Hall et al. (52). In line with other studies reporting a decreased PD1 expression after long-term TILs expansion (53, 54), we observed decreased PD1 expression on TILs after REP in both CD4^+^ and CD8^+^ T cells, probably due to the reduced proliferative potential of exhausted (PD1^+^) T cells (55). On the contrary, Li et al. did not observe any difference in PD1 expression during REP in melanoma (56).

Preclinical and clinical studies (33, 57) have established that the adoptive transfer of early effector T_CM_ CD8^+^ T cells, which possess higher levels of CD27, provides superior immunity compared with the transfer of late antigen-experienced (CD62L^–^CCR7^–^CD27^–^) T_EM_ (33, 58). In our study, we used CD45RA and CD27 markers to determine the subtype of expanded TILs (59, 60). We observed that the CD4^+^ T cells became predominantly T_EM_ after REP, as characterized by downregulation of the CD27 costimulatory marker. On the other hand, the CD8^+^ T cell population acquired expression of CD27 after REP, resulting in a small but significant increase in the central memory phenotype. The latter observation might emerge as contradictory to what has been observed in other TIL expansion studies, as these mostly found losses of CD28 and CD27 after extensive culturing of T cells during REP (57, 61). However, expansion of TILs from different solid tumors might follow different dynamics; hence, reported observations in other studies might not be relevant in GCT.

When multiple independent TIL cultures are generated from a single tumor and screened for tumor recognition, they often exhibit multiple patterns of reactivity (61). In GTC, not all the TIL culture derived from an individual tumor showed the same level of reactivity, but importantly, all the patients demonstrated at least 1 tumor-reactive culture. The reactivity indicates that T cells are functionally active after REP and recognize 1 or more tumor antigen. In some samples, REP increased TILs’ ability to react to the autologous tumor (i.e., patient 2493), a feature likely caused by increased CD8^+^ T cell numbers during REP. By using anti-MHCI blocking antibody, we were able to abrogate T cell activation in some TIL cultures, which indicated that tumor recognition was MHCI mediated. Importantly, tumor cells from patient 2522 successfully grew in vitro and allowed us to assess cytotoxicity of autologous expanded TILs, further validating their functionality. As previously achieved in other malignancies (56), we aimed to define the antigen recognized by T cells. FOXL2 is a marker for granulosa cells (17) and consistently contains a somatic nonsynonymous mutation (1, 2), and its overexpression correlates with worse survival in GCT (23). Hence, we tested the reactivity of TILs against FOXL2-derived peptides, and we observed that 4 of 7 GCT patients showed spontaneous adaptive response against FOXL2, indicating that FOXL2 is a target of GCT. Given that FOXL2 expression has been reported in some breast (21) and cervical (22) cancers, we don’t exclude that, in addition to being a marker and target in GCT, FOXL2 could be a potential target in those malignancies where its expression is elevated. The peptide-based approach facilitates detection of CD8^+^ and CD4^+^ T cells, and it has been suggested to provide higher sensitivity in mapping immune dominant epitopes than current prediction software (62). Our data suggest that GCT is an immunogenic tumor that often encloses functional tumor-reactive FOXL2-specific T cells within the repertoire of expanded TILs.

Having demonstrated that FOXL2 is a shared TAA in GCT and that patients often possess FOXL2-specific T cells, we developed the FoxL2-TT plasmid-DNA vaccine (14) and tested feasibility and efficacy of immunotherapy targeting FOXL2. We fused the mouse *Foxl2* sequence with *TT* to enhance immunogenicity through several possible mechanisms. The whole C fragment of TT activates DCs to secrete cytokines involved in CD4^+^ T cell activation (12, 35). In addition, fragment C contains a universal T helper epitope (p30) effective across different MHCII haplotypes in mice and humans and elicits strong CD4^+^ responses (63). To further enhance the vaccine’s efficacy, injections of the plasmid-DNA were followed by in vivo electroporation, which increases transfection and generates localized inflammation (11), and it has facilitated translation of DNA vaccines in clinical trials (9). Our vaccine also used full-length antigen rather than short peptides, thus including all the possible epitopes present within FOXL2 and bypassing MHC restriction. All these attributes likely contributed to the generation of potent anti-FOXL2 immune responses that we observed targeted against FOXL2 peptide’s pools C and D in C57BL/6, BALB/c, and Tg (HLA-A2.1) mice. Lack of reactivity to FOXL2^C130W^ indicate that the epitopes containing the mutation are not immunogenic in mice, as they are not in human, probably due to the inability of the peptide to strongly bind MHC molecules.

A handful of mouse models of ovarian GCT have been developed and are summarized by Kim et al. (36). As reported, there is not any GCT cell line syngeneic for BALB/c, C57BL/6, or FVB mice that also expresses FOXL2. In order to find suitable models to test our vaccine, we screened several tumor cell lines, and in line with FOXL2 expression restriction in female organs, we found only modest levels of *Foxl2* cDNA in 2 ovarian cancer cell lines (BR5 and ID8) and 1 breast cancer cell line (4T1). The latter observation is in agreement with a study by Wegman et al., who demonstrated that FOXL2 is expressed in some breast cancer patients (21). Because of the very low endogenous level of *Foxl2*, we decided to overexpress the mutated form of *Foxl2* to generate BR5-FOXL2 and 4T1-FOXL2 cell lines. Using a plasmid-DNA vaccine encoding an irrelevant antigen (TEM1) not expressed by 4T1 cells (11), we proved that solely T cells primed by the FoxL2-TT vaccination, and not by irrelevant antigenic portions of the vector, actively recognize the FOXL2-expressing tumor. In the BR5 model, a basal response by FOXL2-restricted T cells against WT cells was observed, likely due to the moderate *Foxl2* cDNA expression in this model. We tested the therapeutic potential of FoxL2-TT vaccination and showed significant tumor control in both tumor models. Moreover, FoxL2-TT vaccination was ineffective against the BR5 WT cell line, proving the specificity of our approach. Immunostaining of FOXL2^+^ tumors in vaccinated mice revealed heavy infiltration of CD8^+^ and CD4^+^ T cells. Given the increased T cell infiltration within the tumor after vaccination, we hypothesized that the vaccine-induced antitumor effect was T cell mediated. To this end, we used adoptive transfer of T cells from vaccinated mice into tumor-bearing mice and showed that a subset of T cells affected tumor progression in BALB/c and C57BL/6 mice. Moreover, we demonstrated that, in preclinical models, ACT was efficacious in controlling tumor progression of established FOXL2-expressing tumors. These findings together with our human TIL results are encouraging and suggest a possible clinical impact of T cell–based therapy in GCT patients. Like human TILs, mouse T cells within the 4T1-FOXL2 tumor express high levels of PD1. Since the PD1/PD-L1 is one of the major axes used by tumors to escape cancer immune attack, we hypothesized that the vaccine’s efficacy could be further improved via combination therapy. Addition of anti–PD-L1 to FoxL2-TT vaccination further suppressed tumor growth and improved mice survival compared with monotherapies. Because vaccination induces reactivity against self-FoxL2 epitopes potentially raising concern about toxicity, we investigate whether FoxL2-TT immunization would alter healthy ovarian follicles or damage reproductive female organs. Notably, FoxL2-TT vaccination does not seem to affect female reproductive system and pregnancy.

Cumulatively, our data underline GCT as immunologically “hot” tumor, heavily infiltrated by T cells, which can be expanded and reinvigorate (64) in vitro, preserving their antitumor activity and their ability to target FOXL2 antigen. Immunization with a plasmid-DNA vaccine encoding murine *Foxl2-tt* confirms immunogenicity of FOXL2 and controls tumor growth of FoxL2-expressing tumors without affecting female reproductive organs. Our preclinical data set in GCT can serve as foundation for clinical development of immunotherapeutic approaches for patient with ovarian GCT

## Methods

### Patients and sample preparation.

Tissues from 11 ovarian GCT patients (Table 1) were collected with informed patient consent at the Penn Medicine Hospital of the University of Pennsylvania. Blood was collected directly into polypropylene tube containing heparin, and PBMCs were separated using Ficol (MilliporeSigma) density gradient centrifugation and cryopreserved in freezing media containing 10% DMSO (Thermo Fisher Scientific) and 90% FBS (Invitrogen). Tumor samples were collected and transported directly to the laboratory for processing. Single tumor cell suspensions from GCT patients were obtained using GentleMACS dissociator (Miltenyi Biotec, 130-093-235), and cell suspensions were cryopreserved in freezing media.

### Animal studies and cell lines.

Six- to 8-week-old FVB (H-2q), C57BL/6 (H-2b), and BALB/c (H-2d) mice were purchased from the Jackson Laboratory. The C57BL/6-Mcph1Tg(HLA-A2.1)1Enge/J mice were purchase from The Jackson Laboratory and bread in-house. ID8 (H-2b), 4T1 (H-2d), RENCA (H-2d), LLC (H-2b), TC1 (H-2b), Hep 1-6 (H-2b), CT26 (H-2d), and OVCAR-5 were cultured in DMEM (Cellgro; 40-101-CV) or in RPMI 1640 (Cellgro; 10-104-CV) medium supplemented with 2 mM L-glutamine (Thermo Fisher Scientific, 35050-061) and 150 U/mL streptomycin plus 200 U/mL penicillin (Cellgro; 30-0010CI), as well as 10% heat-inactivated FBS (Invitrogen, 16000044). Cell lines were obtained from ATCC with the exception of the BR5 (H-2q) cell line, which was donated by Sarah Adams (University of Pennsylvania). Cell were used for 15–20 passages. The GCT cells derived from primary fresh tumor fragments were cultured in DMEM F12 (Thermo Fisher Scientific, 11320033) containing 20% of FBS, 10 mg/mL of ascorbic acid (MilliporeSigma, A4544-25G), 50 ng/mL of hEGF (PeproTech, 100-15), 20 ng/mL of hFGF (PeproTech, 100-18B), and 150 U/mL streptomycin plus 200 U/mL penicillin. GCT identity was routinely tested by sequencing FOXL2 containing the C389G mutation. FOXL2-overexpressing cell lines were obtained upon transfection with Lipofectamine 3000 (Invitrogen, L3000008), selection with 2 μg/mL of puromycin (InvivoGen; ant-pr-1), and isolation of the monoclonal cell population by limiting dilution in 96-well plates. The resulting monoclonal cell lines (BR5-FOXL2 and 4T1-FOXL2) were followed for several passages to determine stable FOXL2 expression, and cells were continuously cultured in media containing 2 μg/mL of puromycin. For in vivo tumor growth experiments, mice were injected using the s.c. route in the lower back with 1 × 10^6^ cells/mouse (BR5 WT and BR5-FOXL2) and 2.5 × 10^5^ cells/mouse (4T1 WT and 4T1-FOXL2). All cell lines were propagated in 5% CO_2_ at 37°C and checked for mycoplasma contamination before tumor challenge.

### Flow cytometry.

Tumor cell suspensions and PBMCs were thawed and stained, and single cell analysis was performed by FACS using 8-parameter flow cytometry on a FACSCanto (BD Biosciences) or 11-parameter flow cytometry on LSRFortessa (BD Biosciences). Expanded TIL samples were stained and analyzed fresh prior to freezing. Immune phenotype of human samples was performed upon staining with the following antibodies: anti-CD45 (BioLegend; clone HI30, 304016), anti-CD3 (BioLegend; clone UCHT1, 300458), anti-CD4 (BD Biosciences; clone RPA-T4, 558116), anti-CD8 (BioLegend; clone SK1, 344714), anti-PD1 (BioLegend; clone EH12.2H7, 329904), anti-CD25 (BioLegend; clone M-A251, 356131), anti-FoxP3 (BioLegend; clone 259D, 320207), anti-CD27 (BioLegend; clone O323, 302819), anti-CD28 (BioLegend; clone CD28.2, 302912), anti-CD45RA (BioLegend; clone HI100, 304125), anti–IFN-γ (eBioscience; clone 4S.B3, 12-7319-42), anti-CD14 (BioLegend; clone M5.E2, 301804), anti-CD11C (BioLegend; clone 3.9, 301614), anti-CD11B (BioLegend; clone ICRF44, 301306), anti–HLA-DR (eBioscience; clone LN3, 45-9956-42), anti-CD15 (BioLegend; clone W6D3, 323033), and anti-CD33 (BioLegend; clone P67.6, 366614), and anti-Lineage (BioLegend; CD3, clone UCHT1; CD14, clone HCD14; CD16, clone 3G8; CD19, clone HIB19; CD20, clone 2H7; CD56, clone HCD56; 348803). For mouse analysis, the following antibodies were used: anti-CD45 (eBioscience; clone 30-F11, 48-0451-82), anti-CD11b (eBioscience; clone M1/70, 12-0112-82), anti-CD11c (eBioscience; clone N418, 69-0114-82), anti–Gr-1 (eBioscience; clone RB6-8C5, 25-5931-82), anti-CD8a (eBioscience; clone 53-6.7, 11-0081-85), anti-CD103 (eBioscience; clone 2E7, 17-1031-82), anti-CD3 (eBioscience; clone 17A2, 46-0032-82), anti-CD4 (eBioscience; clone GK 1.5, 25-0041-82), anti-CD8a (eBioscience; clone 53-6.7, 17-0081-82), anti-CD44 (eBioscience; clone IM7, 47-0441-82), anti-62L (eBioscience; clone MEL-14, 25-0621-82), and anti-PD1 (eBioscience; clone RMP1-30, 46-9981-82). LIVE/DEAD cell stain kit (Invitrogen, L34966) was also used. For staining of FOXL2 overexpression, anti-FOXL2 antibody (Novus, NBP2-22473) was used together with secondary Alexa Fluor 594 antibody anti–rabbit IgG (Invitrogen, A11012). Transcription factor staining buffer set (eBioscience, 00-5523-00) was used to permeabilize cells and stain for all the intracellular markers.

### Initial TIL culture.

TIL expansion was performed as described (50). Briefly, freshly resected GCT were minced into ~1–2 mm^3^ fragments and placed in 24-well plates with 2 mL of TIL complete medium (CM) containing 3000 IU/mL of recombinant human IL-2 (rhIL-2; PEPROTECH), and GCT TILs were allowed to extravasate from the tissue. TIL were expanded in vitro for 3–5 weeks in CM consisting of RPMI 1640, 25 mmol/L HEPES, pH 7.2, 100 U/mL penicillin, 100 μg/mL streptomycin, 2 mmol/l-glutamine, and 5.5 × 10^−5^ mol/L β-mercaptoethanol, supplemented with 10% human serum (Sigma-Aldrich, H4522). Half of the medium was replaced in all wells no later than 1 week after culture initiation and then twice weekly. Cell density was maintained at about 1 × 10^6^ cells/mL, and cells were split into 2 daughter wells when needed. Each initial well was considered an independent TIL culture (fragment) and maintained separately from the others. When individual fragments reached about 2 × 10^6^, FACS analysis was performed and the REP was implemented.

### REP.

REP expansion was performed as previously described (51). The REP used anti-CD3 antibody (Miltenyi Biotec, clone OKT3, 130-093-387) and rhIL-2 in the presence of irradiated, allogeneic feeder cells at a 200:1 ratio of feeder cells to TIL cells. Frozen PBMC feeder cells were obtained each time from 3 different healthy donors, thawed, washed, resuspended in CM, and irradiated (50 Gy). A total of 2 × 10^8^ PBMC, OKT3 antibody (30 ng/mL), CM (75 mL), AIM V media (Thermo Fisher Scientific, 75 mL), and 1 × 10^6^ TIL cells were combined into a 175 cm^2^ tissue culture flask. Flasks were incubated upright at 37°C in 5% CO_2_, and rhIL-2 was added at 3000 IU/mL on day 2. On day 5 and day 10, 120 mL of culture supernatant was removed by aspiration, and media was replaced with a 1:1 mixture of CM/AIM V containing 3000 IU/mL IL-2. REP typically was allowed to proceed for 2 weeks, and cell expansion was monitored throughout. At the end of REP, cells were analyzed phenotypically by FACS, tested functionally by ELISA or ICS, and finally cryopreserved.

### IFN-γ ELISA.

To test TIL reactivity against autologous tumors, cocultures were established at a 1:1 ratio using 3 × 10^5^ to 5 × 10^5^ expanded TILs (fresh, never frozen) and autologous primary tumor cell suspensions that had been cryopreserved in 10% DMSO (Corning) and 90% FBS (Invitrogen, 16000044). To test TIL reactivity against FOXL2, cocultures of rhIL-2–rested TILs (1 × 10^5^) and autologous PBMC (1 × 10^5^) were incubated overnight with 1 μg/mL of individual peptide or peptides pools. For all the assays, TILs (after REP) were allowed to rest from rhIL2 and anti-CD3 stimulation for 7 days before coculture. Single tumor cell suspensions were CD45 depleted using EasySep depletion kit (Stemcell Technologies, 18259) before coculture. Where class I blocking experiments were performed, anti–HLA-ABC (BioLegend, clone W6/W32, 311402) was added to the tumor cells at 10 μg/mL and incubated for 30 minutes at room temperature before setting up the coculture. The incubation was carried out at 37°C overnight before the supernatant was harvested and analyzed for IFN-γ production using ELISA (ELISA MAX, BioLegend, 430104). To improve data presentation, IFN-γ values were normalized by subtracting the negative control (i.e., T cells alone) from the “Tumor + T cells” values or “T cells + PBMCs” values.

### IFN-γ ICS.

A total of 5 × 10^5^ rhIL-2–rested TILs in 1 mL RPMI with 10% FBS was incubated at a 1:1 ratio with autologous PBMCs loaded with one of the peptide pool or single peptide (final concentration 5 μg/mL). For mouse experiments, 1 × 10^6^ splenocytes from vaccinated mice were incubated with peptide pool or single peptide at 5 μg/mL. A total of 0.7 μL/mL of GolgiPlug (BD Biosciences, 555029) was added to the cultures, and incubation was allowed to proceed at 37°C for 12–16 hours. Cells were washed, stained with surface antibodies, fixed and permeabilized with transcription factor staining buffer set (Invitrogen, 00-5523-00), and incubated with anti–hIFN-γ (eBioscience; clone 4S.B3, 12-7319-42) or anti–mouse IFN-γ (eBioscience; clone XMG1.2, 48-7311-82). Cells were analyzed on a FACSCanto flow cytometer using FlowJo software (Tree Star Inc.).

### Cytotoxic assay.

On day 1, target cells (GCT cell line and OVCAR-5) were stained with CellTrace CFSE (Invitrogen) following manufacturer’s recommendation and plated in a 24-well plate at 1 × 10^5^/well. The day afterward, increasing ratios of TILs were added to the wells, and the plate was incubated overnight. On day 3, cells were collected and stained with LIVE/DEAD fixable violet dead cells stain kit (Invitrogen, L34964) and with anti-CD3 (eBioscience; clone 17A2, 46-0032-82). Cells were analyzed on a FACSCanto flow cytometer using FlowJo software.

### IFN-γ ELISpot.

To test T cell reactivity against the target cell lines (BR5-FOXL2 and 4T1-FOXL2), cocultures were established in an ELISpot plate using 5 × 10^5^ splenocytes or 1 × 10^5^ T cells from vaccinated mice, respectively. 4T1-FOXL2 and 4T1 WT were pretreated for 24 hours with 5 ng/mL of rIFN-γ used to increase MHCI expression levels. Cells were then washed twice before starting the coculture. Ninety-six–well MAIP plates (MilliporeSigma; N4510) were coated overnight with a 1:400 dilution in sterile PBS of rat anti–mouse IFN-γ (BD Biosciences; clone R4-6A2, 551216). Splenocytes were plated at 0.1 × 10^6^ to 1 × 10^6^ cells/well and incubated overnight at 37°C with 1 μg/mL peptides. After incubation, plates were washed with PBS and 0.05% Tween-20 (Bio-Rad; 170-6531) and incubated with anti–mouse biotin-conjugated anti–IFN-γ antibody (BD Biosciences; clone R4-6A2, 551506). After washing the plate, Streptavidin-alkaline phosphatase conjugate (BD Biosciences; 554065) was then added for 30 minutes. Plates were developed by adding nitroblue tetrazolium/5-bromo-4-chloro-3-indolyl phosphate (Pierce), and spots were then counted using an automated ELISpot reader (Autoimmun Diagnostika GmbH).

### Real-time PCR.

The relative quantification (65) of the expression levels of selected genes was carried out by real-time PCR using an ABI PRISM Viia7 (Applied Biosystems). Total RNA from tissues was extracted using Trizol reagent (Invitrogen) according to the manufacturer’s instructions. The purity of the RNA samples was determined by visualization of intact 18S and 28S RNA bands in agarose gel electrophoresis. A total of 2 μg of RNA was used for cDNA synthesis using high-capacity cDNA reverse transcription kit (Applied Biosystem, 4368814). cDNA (50 ng) was used in each real-time PCR reaction run. The following TaqMan gene expression assays were used to quantify expression levels of mGapdh (Mm99999915_g1), mFoxl2 (Mm00843544_s1), mFoxl2_opt (ARPRKX6), hGapdh (Hs02786624_g1), hFoxl2 (Hs00846401_s1), and hCD274 (Hs00204257_m1).

### DNA vectors and immunization procedures.

Transfection plasmid was generated by inserting mouse *Foxl2* cDNA (nt 1-1128; aa 1-375) containing the mutation C389G (C130W) into the pCMV6-A-Puro (Origene; PS100025) expression plasmid endowed with puromycin resistance gene. The vaccine plasmid was generated by fusing mutated mouse *Foxl2* cDNA with the cDNA of the aminoterminal domain of fragment C of TT (nt 865–1120). The TT fragment DNA was introduced at the 3′ end of the FoxL2 coding sequence, generating the sequence FoxL2-TT that was then inserted into a pVAX plasmid (Thermo Fisher Scientific; V26020). The TEM1 plasmid DNA was generated by fusing the full-length mouse Tem1 cDNA with a cDNA corresponding to the N-terminal domain of fragment C of TT (865–1120). The TT fragment DNA was introduced at the 3′ end of the Tem1 coding sequence, generating the sequence Tem1-TT, which was then inserted into a pVAX plasmid. The cDNA of Foxl2-TT and Tem1-TT was optimized for codon usage for mouse and synthesized by oligonucleotide assembly (GeneArt; Invitrogen). All constructs were routinely sequenced by the DNA sequencing core facility at University of Pennsylvania. DNA immunization was performed as described previously (11). Briefly, 50 μg plasmid in PBS was injected i.m., and electroporation was performed (2 pulses at 100 mV for 200 ms) immediately after injection.

### In vivo anti-PD-L1 antibody treatment.

The mice received 4 doses (every 3 days) of 200 μg/mouse of anti–PD-L1 (Bio X Cell; clone 10F.9G2, BE0101) blocking antibody by i.p. injection.

### Synthetic peptides.

The mouse and human FOXL2 peptide libraries, each composed by 91 peptides, were synthesized as 15 mers overlapping by 10 aa and purchased from Mimotopes and Eunoia biotech, respectively. The ninety-one 15-mer peptides were dissolved in DMSO at 20 μg/μL and were divided into pools A–D, with approximately 23 peptides each (pool D contained fewer peptides). Pools and individual peptides were used at 1 μg/mL for ELISA and ELISpot and at 5 μg/mL for ICS. Once immunoreactive pools were identified, peptides from pools C and D were tested individually to identify the immunodominant peptides.

### Human protein atlas.

Information regarding FOXL2 distribution on normal organs were collected on 4/26/2020 following this link: https://www.proteinatlas.org/ENSG00000183770-FOXL2/tissue

### MHCI binding prediction method.

The mouse FOXL2 protein sequence (putative forkhead transcription factor [Mus musculus]; AF522275) was fed into the IEDB analysis resource consensus tool on 4/16/2019. H-2Kb allele for C57BL/6 and H-2Kd allele for BALB/c and all peptide length (9-14 aa) were selected for the analysis. We used default settings (recommended) including peptides sorted by percentile rank and IEDB prediction methods.

### Reproduction and histologic evaluation.

To test whether FoxL2-TT immunization affects mouse pregnancy by interfering with the reproductive organs, female mice were immunized 3 times, and 1 week after the last immunization, mice were allowed to mate with individually housed males. Coitus was monitored every day and confirmed by the presence of a vaginal plug. Time to gestation, pup weight at birth, and total litter size were measured at each birth over 4 rounds of pregnancy. In another experiment, mice were challenged with 4T1-FOXL2 and vaccinated 3 times as described earlier. One week after the last immunization, mice were sacrificed, and ovary, fallopian tube, and uterus were harvested and fixed in 4% paraformaldehyde. Tissues were embedded in paraffin and stained at the skin biology and diseases resource-based center (SBDRC) at the University of Pennsylvania. Histopathologic evaluation was performed on standard H&E sections.

### Adoptive transfer of cells.

ACT injections were performed as described (66). Tumor-free mice were vaccinated 3 times with FoxL2-TT and sacrificed 1 week later. CD3^+^, CD4^+^, and CD8^+^ T cells were magnetically isolated from the spleens. Isolated T cells were injected i.v. (1 × 10^7^ CD3^+^, 6 × 10^6^ CD4^+^, and 4 × 10^6^ CD8^+^) into tumor-bearing mice (challenged 2 days before transfer) that had been sublethally irradiated (400 rads) 1 day before tumor challenge.

### WB.

WB was performed with total cell lysate using antibody specific for FOXL2 (Abcam, ab5096), β-actin rabbit antibody (Cell Signaling Technology, 064967S). Membranes were incubated with a 1:2000 dilution of HRP-conjugated antibody rabbit to goat IgG (Abcam; ab97100) and anti-rabbit IgG (Cell Signaling Technology; 7074P2) and developed with the ECL system.

### Statistics.

For comparisons of more than 2 groups, we used Tukey’s multiple comparison tests. For all other comparisons, 2-tailed Student’s *t* tests using a pooled estimate of the variance were used. For all the mice experiments, sample sizes were chosen based on pilot experiments and our experience with similar experiments. For tumor progression experiments, we used the 2-way ANOVA (or mixed model) and the 2-tailed Student’s *t* tests. *P < 0.05, **P < 0.01, ***P < 0.001.

### Study approval.

All animal studies were approved by the IACUC and University Laboratory Animal Resources at the University of Pennsylvania. Mice were treated in accordance with University of Pennsylvania guidelines. Patients were collected under a research protocol that was approved by the University of Pennsylvania IRB.

## Author contributions

SP helped design the studies, performed the experiments, analyzed the data, and drafted the manuscript. JLT provided human GCT samples and edited the manuscript. FS provided GCT samples and edited the manuscript. EG helped with patients and edited the manuscript. MUH performed experiments. RD assisted in study design and edited the manuscript. RB assisted patients and edited the manuscript. MAM helped in conceiving the project and writing the manuscript. AF conceived the experiments, supervised the project, and wrote the manuscript.

## Supplementary Material

Supplemental data

## Figures and Tables

**Table 1:**
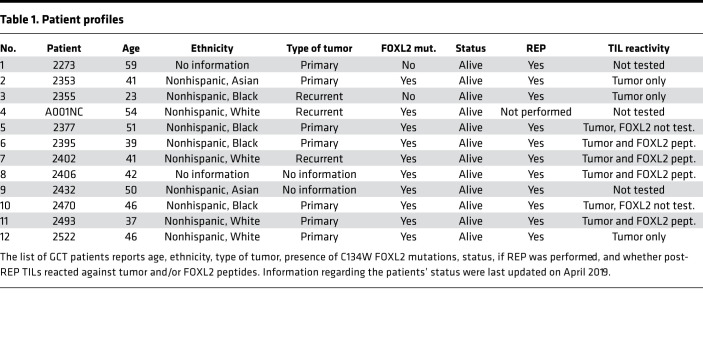
Patient profiles

## Version 1 08/20/2020

Electronic publication

## References

[1] Köbel M, Gilks CB, Huntsman DG (2009). Adult-type granulosa cell tumors and FOXL2 mutation. Cancer Res.

[2] Kim SY (2016). Constitutive Activation of PI3K in Oocyte Induces Ovarian Granulosa Cell Tumors. Cancer Res.

[3] Pierini S (2015). A Tumor Mitochondria Vaccine Protects against Experimental Renal Cell Carcinoma. J Immunol.

[4] Uribe-Herranz M (2018). Gut microbiota modulates adoptive cell therapy via CD8+ dendritic cells and IL-12. JCI Insight.

[5] Rosenberg SA (2011). Durable complete responses in heavily pretreated patients with metastatic melanoma using T-cell transfer immunotherapy. Clin Cancer Res.

[6] Kalos M (2011). T cells with chimeric antigen receptors have potent antitumor effects and can establish memory in patients with advanced leukemia. Sci Transl Med.

[7] Dudley ME (2013). Randomized selection design trial evaluating CD8+-enriched versus unselected tumor-infiltrating lymphocytes for adoptive cell therapy for patients with melanoma. J Clin Oncol.

[8] Haskins-Coulter T, Southern J, Andrews N, Miller E (2017). Reactogenicity of Cervarix and Gardasil human papillomavirus (HPV) vaccines in a randomized single blind trial in healthy UK adolescent females. Hum Vaccin Immunother.

[9] Pierini S (2017). Trial watch: DNA-based vaccines for oncological indications. Oncoimmunology.

[10] Pol J (2015). Trial Watch: Peptide-based anticancer vaccines. Oncoimmunology.

[11] Facciponte JG (2014). Tumor endothelial marker 1-specific DNA vaccination targets tumor vasculature. J Clin Invest.

[12] Facciabene A (2006). DNA and adenoviral vectors encoding carcinoembryonic antigen fused to immunoenhancing sequences augment antigen-specific immune response and confer tumor protection. Hum Gene Ther.

[13] Stevenson FK, Rice J, Ottensmeier CH, Thirdborough SM, Zhu D (2004). DNA fusion gene vaccines against cancer: from the laboratory to the clinic. Immunol Rev.

[14] Rice J, Elliott T, Buchan S, Stevenson FK (2001). DNA fusion vaccine designed to induce cytotoxic T cell responses against defined peptide motifs: implications for cancer vaccines. J Immunol.

[15] Bellessort B (2015). Role of Foxl2 in uterine maturation and function. Hum Mol Genet.

[16] Crisponi L (2001). The putative forkhead transcription factor FOXL2 is mutated in blepharophimosis/ptosis/epicanthus inversus syndrome. Nat Genet.

[17] Shah SP (2009). Mutation of FOXL2 in granulosa-cell tumors of the ovary. N Engl J Med.

[18] [No authors listed]. Tissue expression of FOXL2 - Summary. The Human Protein Atlas. https://www.proteinatlas.org/ENSG00000183770-FOXL2/tissue Accessed July 29, 2020

[19] Egashira N, Takekoshi S, Takei M, Teramoto A, Osamura RY (2011). Expression of FOXL2 in human normal pituitaries and pituitary adenomas. Mod Pathol.

[20] Rosario R, Cohen PA, Shelling AN (2014). The role of FOXL2 in the pathogenesis of adult ovarian granulosa cell tumours. Gynecol Oncol.

[21] Wegman P, Göthlin Eremo A, Lindlöf A, Karlsson M, Stål O, Wingren S (2011). Expression of the forkhead transcription factor FOXL2 correlates with good prognosis in breast cancer patients treated with tamoxifen. Int J Oncol.

[22] Liu XL, Meng YH, Wang JL, Yang BB, Zhang F, Tang SJ (2014). FOXL2 suppresses proliferation, invasion and promotes apoptosis of cervical cancer cells. Int J Clin Exp Pathol.

[23] D’Angelo E (2011). Prognostic significance of FOXL2 mutation and mRNA expression in adult and juvenile granulosa cell tumors of the ovary. Mod Pathol.

[24] Schmidt D (2004). The murine winged-helix transcription factor Foxl2 is required for granulosa cell differentiation and ovary maintenance. Development.

[25] Fang H, Declerck YA (2013). Targeting the tumor microenvironment: from understanding pathways to effective clinical trials. Cancer Res.

[26] Ahmadzadeh M (2009). Tumor antigen-specific CD8 T cells infiltrating the tumor express high levels of PD-1 and are functionally impaired. Blood.

[27] Maine CJ (2014). Programmed death ligand-1 over-expression correlates with malignancy and contributes to immune regulation in ovarian cancer. Cancer Immunol Immunother.

[28] Sato E (2005). Intraepithelial CD8+ tumor-infiltrating lymphocytes and a high CD8+/regulatory T cell ratio are associated with favorable prognosis in ovarian cancer. Proc Natl Acad Sci USA.

[29] Elliott LA, Doherty GA, Sheahan K, Ryan EJ (2017). Human Tumor-Infiltrating Myeloid Cells: Phenotypic and Functional Diversity. Front Immunol.

[30] Bronte V (2016). Recommendations for myeloid-derived suppressor cell nomenclature and characterization standards. Nat Commun.

[31] Topalian SL, Muul LM, Solomon D, Rosenberg SA (1987). Expansion of human tumor infiltrating lymphocytes for use in immunotherapy trials. J Immunol Methods.

[32] Jin J (2012). Simplified method of the growth of human tumor infiltrating lymphocytes in gas-permeable flasks to numbers needed for patient treatment. J Immunother.

[33] Gattinoni L (2005). Acquisition of full effector function in vitro paradoxically impairs the in vivo antitumor efficacy of adoptively transferred CD8+ T cells. J Clin Invest.

[34] Di Mitri D (2011). Reversible senescence in human CD4+CD45RA+CD27- memory T cells. J Immunol.

[35] Rice J, Ottensmeier CH, Stevenson FK (2008). DNA vaccines: precision tools for activating effective immunity against cancer. Nat Rev Cancer.

[36] Kim SY (2016). Insights into granulosa cell tumors using spontaneous or genetically engineered mouse models. Clin Exp Reprod Med.

[37] Lei J (2017). Maternal dendrimer-based therapy for inflammation-induced preterm birth and perinatal brain injury. Sci Rep.

[38] Lei J (2018). Maternal CD8^+^ T-cell depletion alleviates intrauterine inflammation-induced perinatal brain injury. Am J Reprod Immunol.

[39] Chaudhary B, Elkord E (2016). Regulatory T Cells in the Tumor Microenvironment and Cancer Progression: Role and Therapeutic Targeting. Vaccines (Basel).

[40] Veglia F, Perego M, Gabrilovich D (2018). Myeloid-derived suppressor cells coming of age. Nat Immunol.

[41] Ugolini A (2020). Polymorphonuclear myeloid-derived suppressor cells limit antigen cross-presentation by dendritic cells in cancer. JCI Insight.

[42] Finke J, Ko J, Rini B, Rayman P, Ireland J, Cohen P (2011). MDSC as a mechanism of tumor escape from sunitinib mediated anti-angiogenic therapy. Int Immunopharmacol.

[43] Sun HL (2012). Increased frequency and clinical significance of myeloid-derived suppressor cells in human colorectal carcinoma. World J Gastroenterol.

[44] Uribe-Herranz M (2020). Gut microbiota modulate dendritic cell antigen presentation and radiotherapy-induced antitumor immune response. J Clin Invest.

[45] Veglia F (2017). Lipid bodies containing oxidatively truncated lipids block antigen cross-presentation by dendritic cells in cancer. Nat Commun.

[46] Zhang Z (2015). Infiltration of dendritic cells and T lymphocytes predicts favorable outcome in epithelial ovarian cancer. Cancer Gene Ther.

[47] Takahashi A (2002). Correlation of vascular endothelial growth factor-C expression with tumor-infiltrating dendritic cells in gastric cancer. Oncology.

[48] Dudley ME (2013). Randomized selection design trial evaluating CD8+-enriched versus unselected tumor-infiltrating lymphocytes for adoptive cell therapy for patients with melanoma. J Clin Oncol.

[49] Radvanyi LG (2012). Specific lymphocyte subsets predict response to adoptive cell therapy using expanded autologous tumor-infiltrating lymphocytes in metastatic melanoma patients. Clin Cancer Res.

[50] Dudley ME, Wunderlich JR, Shelton TE, Even J, Rosenberg SA (2003). Generation of tumor-infiltrating lymphocyte cultures for use in adoptive transfer therapy for melanoma patients. J Immunother.

[51] Baldan V, Griffiths R, Hawkins RE, Gilham DE (2015). Efficient and reproducible generation of tumour-infiltrating lymphocytes for renal cell carcinoma. Br J Cancer.

[52] Hall M (2016). Expansion of tumor-infiltrating lymphocytes (TIL) from human pancreatic tumors. J Immunother Cancer.

[53] Gros A (2014). PD-1 identifies the patient-specific CD8+tumor-reactive repertoire infiltrating human tumors. J Clin Invest.

[54] Mujib S (2012). Antigen-independent induction of Tim-3 expression on human T cells by the common γ-chain cytokines IL-2, IL-7, IL-15, and IL-21 is associated with proliferation and is dependent on the phosphoinositide 3-kinase pathway. J Immunol.

[55] Wei F (2013). Strength of PD-1 signaling differentially affects T-cell effector functions. Proc Natl Acad Sci USA.

[56] Li Y, Liu S, Hernandez J, Vence L, Hwu P, Radvanyi L (2010). MART-1-specific melanoma tumor-infiltrating lymphocytes maintaining CD28 expression have improved survival and expansion capability following antigenic restimulation in vitro. J Immunol.

[57] Powell DJ, Dudley ME, Robbins PF, Rosenberg SA (2005). Transition of late-stage effector T cells to CD27+ CD28+ tumor-reactive effector memory T cells in humans after adoptive cell transfer therapy. Blood.

[58] Klebanoff CA, Gattinoni L, Restifo NP (2012). Sorting through subsets: which T-cell populations mediate highly effective adoptive immunotherapy?. J Immunother.

[59] Harari A, Vallelian F, Pantaleo G (2004). Phenotypic heterogeneity of antigen-specific CD4 T cells under different conditions of antigen persistence and antigen load. Eur J Immunol.

[60] Appay V, van Lier RA, Sallusto F, Roederer M (2008). Phenotype and function of human T lymphocyte subsets: consensus and issues. Cytometry A.

[61] Tran KQ (2008). Minimally cultured tumor-infiltrating lymphocytes display optimal characteristics for adoptive cell therapy. J Immunother.

[62] Wu C (2011). Systematic identification of immunodominant CD8+ T-cell responses to influenza A virus in HLA-A2 individuals. Proc Natl Acad Sci USA.

[63] Panina-Bordignon P, Tan A, Termijtelen A, Demotz S, Corradin G, Lanzavecchia A (1989). Universally immunogenic T cell epitopes: promiscuous binding to human MHC class II and promiscuous recognition by T cells. Eur J Immunol.

[64] Wherry EJ, Kurachi M (2015). Molecular and cellular insights into T cell exhaustion. Nat Rev Immunol.

[65] Pierini S (2014). Promoter hypermethylation of CDKN2A, MGMT, MLH1, and DAPK genes in laryngeal squamous cell carcinoma and their associations with clinical profiles of the patients. Head Neck.

[66] Facciabene A (2017). Local endothelial complement activation reverses endothelial quiescence, enabling t-cell homing, and tumor control during t-cell immunotherapy. Oncoimmunology.

