# Supplementary material for: Ovarian granulosa cell tumor characterization identifies FOXL2 as an immunotherapeutic target
Source: JCI Insight. 2020 Aug 20;5(16):e136773. doi: 10.1172/jci.insight.136773 (PMC7455139; doi:10.1172/jci.insight.136773)
Supplement: Supplemental data [file jciinsight-5-136773-s174.pdf]

A

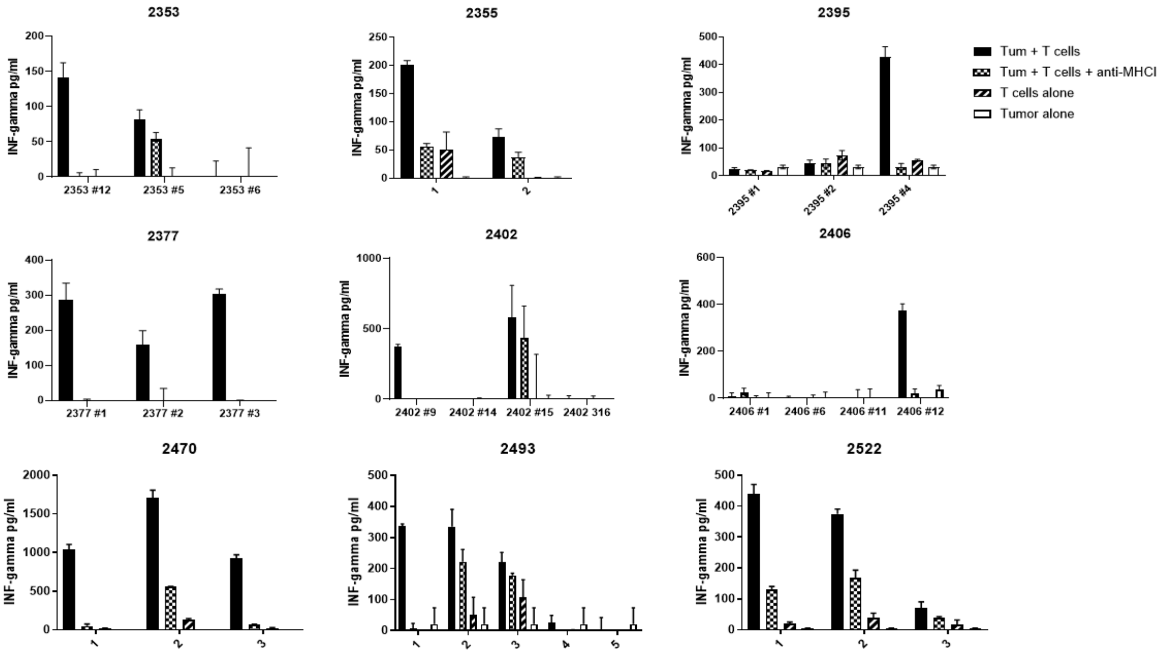

B

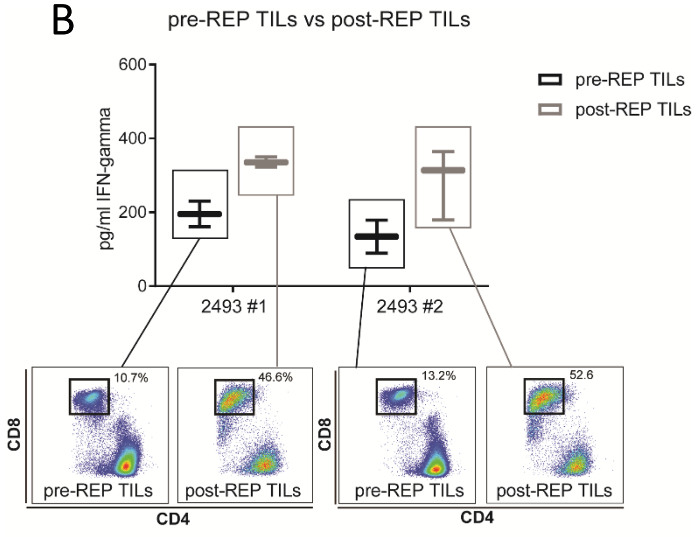

Figure Supplementary 1. (A) Expanded TILs recognize autologous tumors. 3-5 × 10^5 of IL-2-rested TILs (post-REP) from individual cultures were co-culture overnight with viable autologous tumor cells (CD45-depleted) at 1:1 ratio. Where class I blocking experiments were performed, anti-HLA-ABC was also added to tumor cells. T cells activation was assessed by measuring secreted IFNγ in the supernatant using ELISA.

The bar charts illustrate the IFNγ values from all patients. Responding TILs fragments are: 2353#12; 2355#1; 2395#3; 2377#1, #2, #3; 2402#9; 2406#12; 2470#1, #2, #3; 2493#1; 2522#1, #2. (B) TILs post-REP show increased activation against target compared to TILs pre-REP. TILs pre- and post-REP were co-cultured with autologous tumor cells and IFNγ levels measured by ELISA. Graph shows normalized values of two representative TILs fragments for patient 2493. Normalized values were obtained by subtracting "T cells alone" value from "T cells + Tumor" values. Flow cytometry show changes in percentage of $CD8^{+}$T cells pre- and post-REP (lower panel).

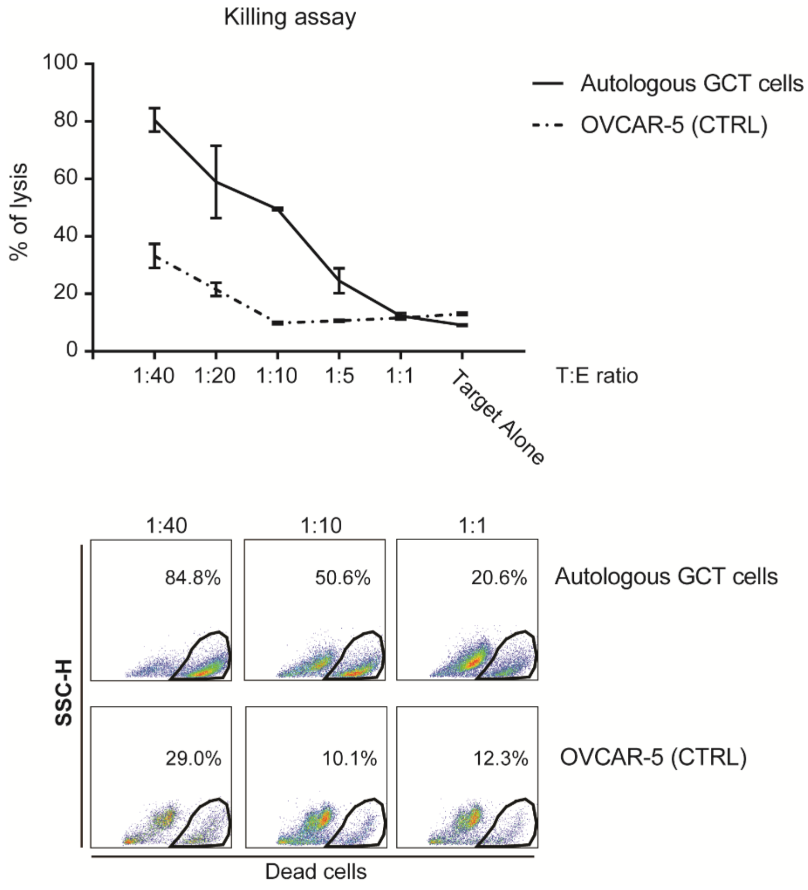

Figure Supplementary 2. GCT TILs are functionally active and kill target cells. Post-REP TILs from patient 2522 were co-cultured overnight with the GCT cell line from patient 2522 and a control cell line (OVCAR-5) and, cytotocity was evaluated by LIVE/DEAD staining and FACS analysis. Graph shows mean values with standard error of % of target cells lysis. Flow cytometry shows proportion of Dead cells in selected representative samples.

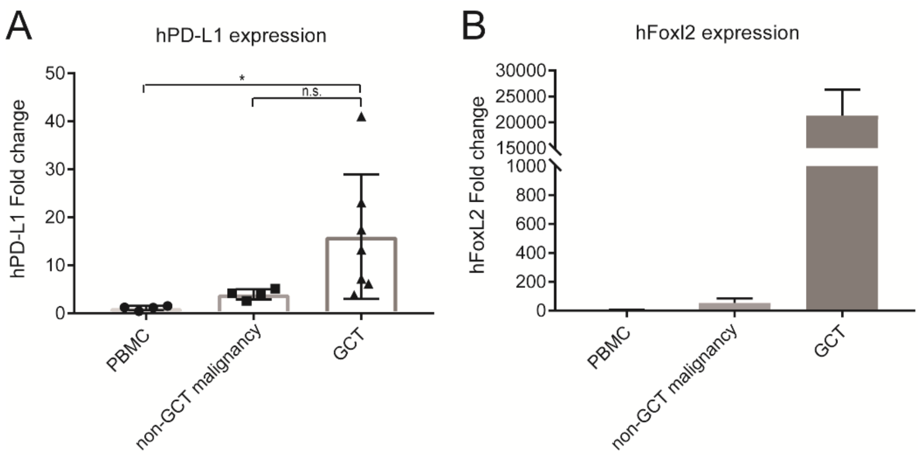

Figure Supplementary 3. PD-L1 and FOXL2 expression levels in ovarian GCT. Bar graphs represent fold-change quantification of endogenous CD274 (PD-L1) (A) and Foxl2 (B) cDNA obtained from flash-frozen PBMCs (n=4), non-GCT malignancy (n=4) and GCT (n=7-8). Means ± SEM are shown. Multiple *t* test analysis was performed. *$P < 0.05$, n.s. non significant.

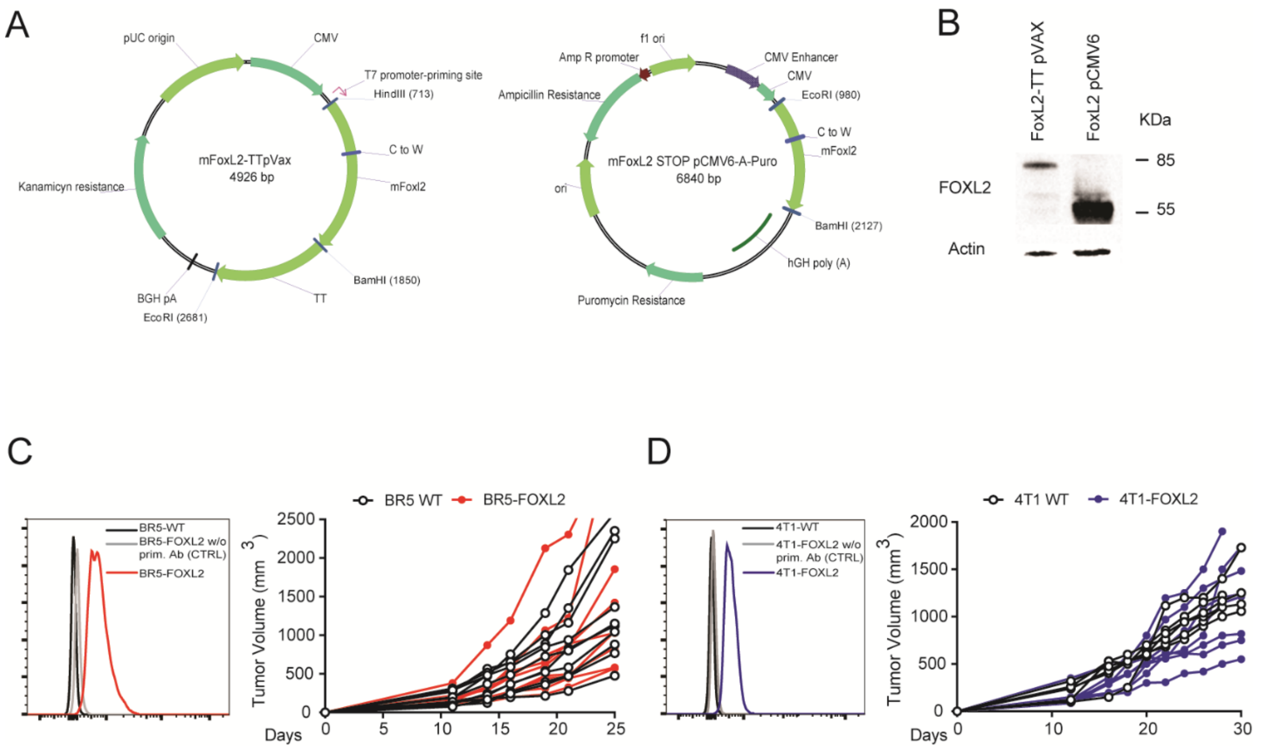

Figure Supplementary 4. Vaccine, vector and FOXL2-expressing cell lines generation. (A) Schematic map of FoxL2-TT vaccine (left panel) and FoxL2_pCMV6-A-Puro (right panel). (B) Western bloat showing sizes of FOXL2-TT fusion protein (~85KDa) and FOXL2 protein (~55KDa). (C and D, left panel) FACS histogram showing stable over-expression of FOXL2 protein in BR5-FOXL2 (read) and 4T1-FOXL2 (blue) cell lines. The black lines represent the BR5 WT and 4T1 WT, the grey lines represent the FOXL2-expressing cell lines without the primary Ab and the red lines represent over-expression of FOXL2. (C and D, right panel) FVB and BALB/c mice were injected s.c. with 1×10^6 of BR5 WT (n=9 mice) and BR5-FOXL2 (n=9 mice) (C) and 2.5×10^5 of 4T1 WT (n=6 mice) and 4T1-FOXL2 (n=7 mice) (D). Each line represents an individual mouse; data are from 1 of 2 experiments.

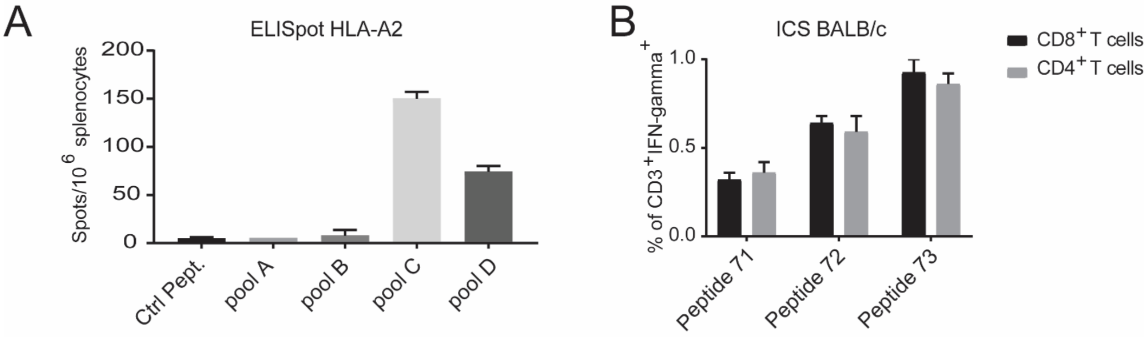

Figue Supplementary 5. Characterization of immune response against FOXL2. 1×10^6 splenocytes from FoxL2-TT vaccinated Tg(HLA-A2.1) and BALB/c mice were stimulated overnight with the mouse FOXL2 library or individual peptide and tested by ELISpot and ICS. Bar charts illustrating number of IFNγ spots (A) for Tg(HLA-A2.1) mice. Percentages of IFNγ-secreting T cells (B) show that both $CD4^+$ and $CD8^+$T cells in BALB/c reacted upon overnight stimulation with peptide #72 and #73 but not against #71. Data are from 1 of 3 independent experiments.

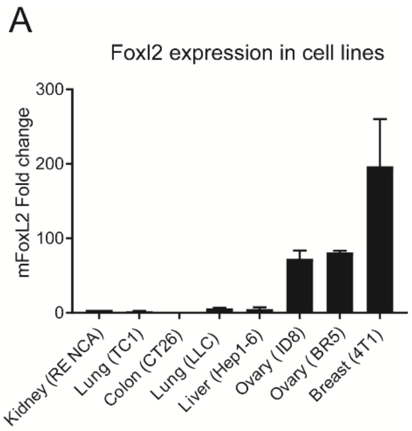

Figure Supplementary 6. Endogenous expression of murine *Foxl2*. (A) Cell lines were allowed to grow in 5% CO_2 at 37 °C and RNA was extracted at passage number 3. Results represent the Means ± SEM of three experiments.

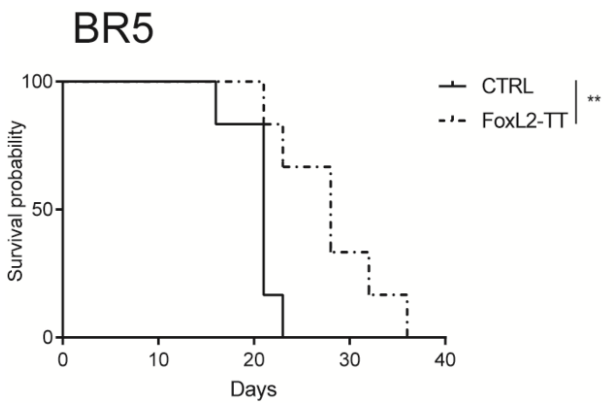

Figure Supplementary 7. FoxL2-TT vaccination extend mice survival. FVB (n=6 mice/group) and BALB/c (n=7 mice/group) were injected s.c. with 1×10^6 BR5-FOXL2 and 2.5×10^5 4T1-FOXL2 and 3 days later injected 3 times with FoxL2-TT or empty pVAX (CTRL) vaccines followed by electroporation. For the Kaplan-Meier analysis, cut-off values for tumor volume of 1400mm3 for BR5; 1000 mm3 for 4T1 were set to estimate mice expiration. Long-rank test was performed to estimate statistical significance. **P* < 0.05, ***P* < 0.01.

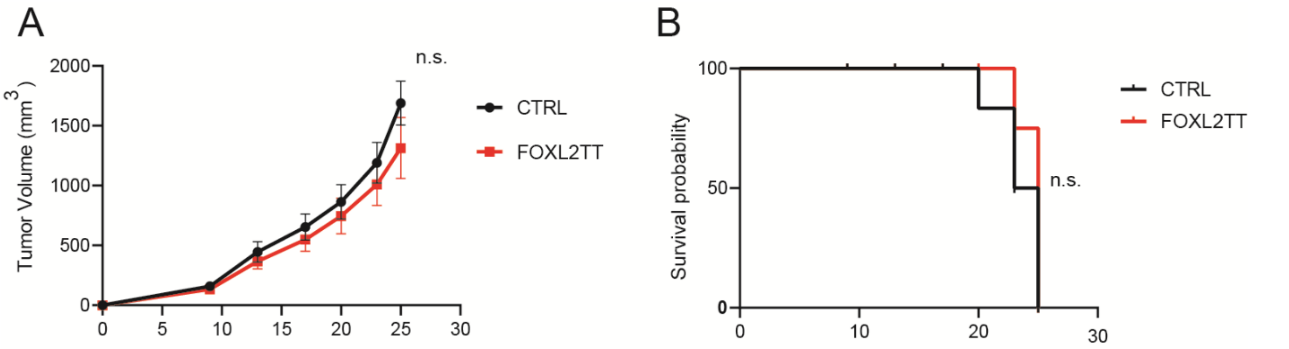

Figure Supplementary 8. FoxL2-TT vaccination is ineffective in BR5-WT model. FVB were injected s.c. with 1×10^6 BR5-WT and 3 days later injected 3 times with FoxL2-TT or empty pVAX (CTRL) vaccines followed by electroporation. To estimate mice expiration in the Kaplan-Meier analysis, the tumor volume's cut-off value was set at 1400mm3. Two-way ANOVA and long-rank test was performed to estimate statistical significance. n.s. Non significant.

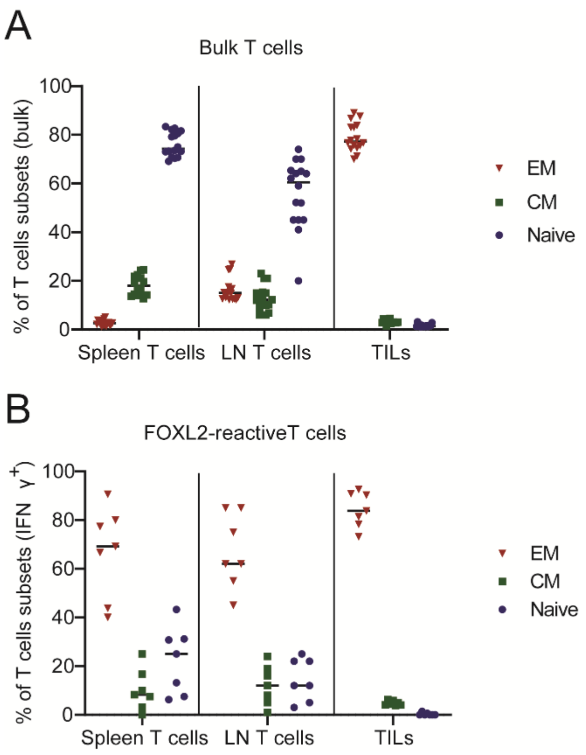

Figure supplementary 9. Characterization of bulk and antigen-specific T cells subsets. BALB/c mice were challenged with 4T1-FOXL2 tumor and vaccinated. Spleen, LN and tumor were collected, and single cells suspension were obtained with mechanical and enzymatic digestion. To determine T cells phenotype, CD44 and CD62L antibody were used on bulk $CD8^+$ T cells (A) or on IFNγ-positive $CD8^+$ T cells (B). IFNγ was detected via ICS after stimulation with peptide #73. Effector memory (EM) cells are $CD62L^-CD44^+$; central memory (CM) cells are $CD62L^+CD44^+$ and naïve cells are $CD62L^+CD44^-$. At least 5 mice where used.

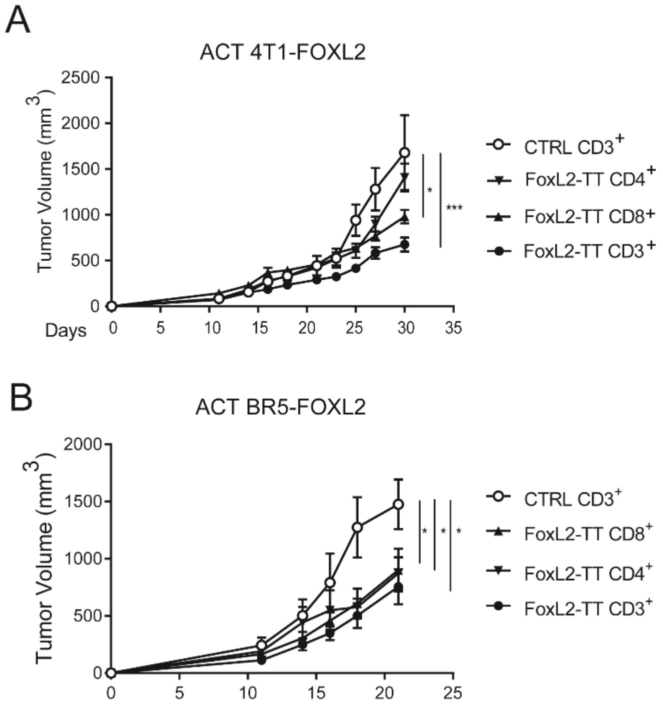

Figure supplementary 10. FoxL2-TT effect is T cells-mediated. FoxL2-TT vaccinated BALB/c mice were sacrificed one week after vaccination and $CD3^+$, $CD8^+$, $CD4^+$T cells were harvested. 4T1-FOXL2 and BR5-FOXL2 tumor bearing mice were sublethally irradiated (400rads) 1 day before tumor challenge and $CD3^+$T cells (10^7cells/mouse), $CD8^+$T cells (4 x 10^6cells/mouse) and $CD4^+$T cells (6 x 10^6cells/mouse) were adoptively transfer (i.v.) two days after tumor challenge (A and B). Control $CD3^+$T cells, from non vaccinated mice, were also used. Data are mean ± SD (n = 5-6 mice per group). 2-tailed t test analyses were performed at day 30 after tumor challenge. *P < 0.05, **P < 0.01, ***P < 0.001, n.s. non significant.

allele	seq_num	start	end	length	peptide	method	Percentile	ann_ic50	ann_rank	smm_ic50
H-2-Kb	1	249	257	9	ASYGPYSRV	Consensus (ann/smm)	0.13	19.63	0.06	58.36
H-2-Kb	1	266	273	8	VVNSYNGL	Consensus (ann/smm)	0.2	42.59	0.11	49.86
H-2-Kb	1	249	262	14	ASYGPYSRVQSMAL	ann	0.43	198.96	0.43	-
H-2-Kb	1	53	61	9	YSYVALIAM	Consensus (ann/smm)	0.43	117.53	0.27	178.69
H-2-Kb	1	137	145	9	GNYRRRRRM	Consensus (ann/smm)	0.82	494.62	0.95	209.95
H-2-Kb	1	245	257	13	AGPAASYGPYSRV	ann	0.93	482.81	0.93	-
H-2-Kb	1	244	257	14	LAGPAASYGPYSRV	ann	0.94	485.5	0.94	-
H-2-Kb	1	249	260	12	ASYGPYSRVQSM	ann	1.2	549.81	1.2	-
H-2-Kb	1	246	257	12	GPAASYGPYSRV	ann	1.3	628.42	1.3	-
H-2-Kb	1	366	373	8	TGALHSRL	Consensus (ann/smm)	1.35	727.06	1.5	259.26
H-2-Kb	1	248	260	13	AASYGPYSRVQSM	ann	1.7	892.47	1.7	-
H-2-Kb	1	51	58	8	PPYSYVAL	Consensus (ann/smm)	1.7	1390.27	2.5	187.39
H-2-Kb	1	249	261	13	ASYGPYSRVQSMA	ann	1.9	975.16	1.9	-
H-2-Kb	1	247	260	14	PAASYGPYSRVQSM	ann	2	1064.46	2	-
H-2-Kb	1	245	258	14	AGPAASYGPYSRVQ	ann	2.1	1128.93	2.1	-
H-2-Kb	1	247	258	12	PAASYGPYSRVQ	ann	2.3	1274.19	2.3	-
H-2-Kb	1	56	63	8	VALIAMAI	Consensus (ann/smm)	2.35	777.87	1.5	821.75
H-2-Kb	1	248	259	12	AASYGPYSRVQS	ann	2.4	1301.29	2.4	-
H-2-Kb	1	246	258	13	GPAASYGPYSRVQ	ann	2.5	1384.73	2.5	-
H-2-Kb	1	248	261	14	AASYGPYSRVQSMA	ann	2.6	1507.42	2.6	-
H-2-Kb	1	254	262	9	YSRVQSMAL	Consensus (ann/smm)	2.6	750.75	1.5	859.23
H-2-Kb	1	265	273	9	GVVNSYNGL	Consensus (ann/smm)	2.6	1431.51	2.5	642.85
H-2-Kb	1	247	259	13	PAASYGPYSRVQS	ann	2.9	1679.68	2.9	-
H-2-Kb	1	73	80	8	LSGIYQYI	Consensus (ann/smm)	2.95	1158.07	2.1	1006.33
H-2-Kb	1	48	59	12	AQKPPYSYVALI	ann	3	1770.14	3	-
H-2-Kb	1	133	145	13	MFEKGNYRRRRRM	ann	3	1736.39	3	-
H-2-Kb	1	75	84	10	GIYQYIIAKF	Consensus (ann/smm)	3	3252.29	5	2554.64
H-2-Kb	1	193	202	10	SGFLNNSWPL	Consensus (ann/smm)	3.05	1768.99	3	3822.35
H-2-Kb	1	246	259	14	GPAASYGPYSRVQS	ann	3.1	1812.35	3.1	-
H-2-Kb	1	250	257	8	SYGPYSRV	Consensus (ann/smm)	3.55	1037.06	2	1431.33
H-2-Kb	1	357	369	13	CSYWDHDSKTGAL	ann	3.6	2153.24	3.6	-
H-2-Kb	1	48	61	14	AQKPPYSYVALIAM	ann	3.7	2234.39	3.7	-
H-2-Kb	1	249	258	10	ASYGPYSRVQ	Consensus (ann/smm)	3.84	358.01	0.69	5252.06
H-2-Kb	1	134	145	12	FEKGNYRRRRRM	ann	4.1	2549.1	4.1	-
H-2-Kb	1	180	191	12	DGYGYLAPPKYL	ann	4.1	2542.46	4.1	-
H-2-Kb	1	50	61	12	KPPYSYVALIAM	ann	4.3	2668.13	4.3	-
H-2-Kb	1	132	145	14	DMFEKGNYRRRRRM	ann	4.3	2723.64	4.3	-
H-2-Kb	1	47	59	13	PAQKPPYSYVALI	ann	4.4	2789.79	4.4	-
H-2-Kb	1	49	61	13	QKPPYSYVALIAM	ann	4.7	2962.03	4.7	-

Supplementary Table 1. Analysis of H-2Kb-restricted binders (9-14 aa length) for mouse FOXL2 protein. The first 40 peptides are shown in the table generated using the IEDB analysis resource consensus tool. Peptides are sorted by Percentile Rank (up to bottom). The predicted output is given in units of IC_{50}nM. A lower number indicates higher affinity. Peptides with IC_{50} values <50 nM are considered high affinity, <500 nM intermediate affinity and <5000 nM low affinity.
